# Decreased PP2A expression and activity represent a therapeutic target for plexiform neurofibroma

**DOI:** 10.1186/s40478-026-02315-w

**Published:** 2026-05-11

**Authors:** Minghui Yue, Yixiao Wang, Jiabao Gu, Cheng Zhang, Yang Chen, Aruna Gao, Kwangmin Choi, Zhaoming Wu, Zhichao Wang, Jay Pundavela, Nancy Ratner, Yanan Yu

**Affiliations:** 1 Xuzhou Medical University, Xuzhou, China; 2 Affiliated Stomatological Hospital of Xuzhou Medical University, Jiangsu Province 221004 Xuzhou, China; 3 Division of Experimental Hematology and Cancer Biology, Cincinnati Children’s Hospital Medical Center, Cincinnati, OH USA; 4 Division of Applied Oral Sciences and Community Dental Care, Faculty of Dentistry, The University of Hong Kong, Hong Kong, China; 5 Neurofibromatosis Type 1 Center and Laboratory for Neurofibromatosis Type 1 Research, Shanghai Ninth People’s Hospital, Shanghai Jiao Tong University School of Medicine, Shanghai, China; 6 Jiangsu Engineering Center for Precision Diagnosis and Treatment Research of Polygenic Diseases, Key Laboratory of Genetic Foundation and Clinical Application, Xuzhou, 221004 Jiangsu Province China; 7 Department of Plastic and Reconstructive Surgery, Shanghai Ninth People’s Hospital, Shanghai Jiao Tong University School of Medicine, Shanghai, 200011 China

**Keywords:** Neurofibromatosis type 1, Neurofibroma, PP2A phosphatase, FTY720

## Abstract

In neurofibromatosis type 1 (NF1), loss-of-function mutations in the *NF1* gene increase activation of the RAS–MEK–ERK signaling cascade, driving tumorigenesis. MEK inhibitors (MEKi) inhibit tumor growth and significantly shrink nerve tumors (neurofibromas). However, MEKi treatment alone fails to eradicate tumor cells, and tumor regrowth occurs after drug withdrawal, highlighting the limitations of targeting the single MEK pathway. An alternative strategy is to promote dephosphorylation of hyperactive kinases that drive tumor growth by enhancing phosphatase activity. We identified deregulated expression of genes encoding subunits of the PP2A phosphatase in neurofibroma and neurofibroma Schwann cells. We confirmed significant reductions in both the expression and enzymatic activity of the PP2A A and C subunits. FTY720, a compound known to restore PP2A phosphatase activity, inhibited tumor sphere formation by mouse and human neurofibroma Schwann cell progenitor cells, suppressed the proliferation of both primary and immortalized neurofibroma-derived Schwann cells, and induced cell apoptosis in vitro. Furthermore, treatment with FTY720—alone or in combination with MEKi—significantly suppressed tumor number and reduced tumor burden in remaining tumors in a murine model of NF1, highlighting the promise of using FTY720 as a novel therapeutic strategy in NF1.

**Supplementary Information:**

The online version contains supplementary material available at 10.1186/s40478-026-02315-w.

## Introduction

Neurofibromatosis type 1 (NF1) is an autosomal-dominant disorder caused by loss-of-function mutations in the *NF1* tumor suppressor gene, which has an estimated incidence of 1 in 3000 individuals worldwide [1–3]. The disease is characterized by highly variable clinical features, including café-au-lait spots, Lisch nodules, bone malformation, learning defects, and the development of benign and malignant tumors, particularly optical gliomas (OPGs), dermal neurofibromas (DNs), plexiform neurofibromas (PNs), and malignant peripheral nerve sheath tumors (MPNSTs) [4, 5]. Around fifty percent of NF1 patients develop PNs in deep nerves, which may induce morbidity in the patients. PNs are driven by biallelic loss of function in tumor Schwann cells (SCs). These tumors also contain fibroblasts, macrophages, dendritic cells, and T cells that can contribute to neurofibroma growth [6, 7]. PNs can transform into MPNSTs, sarcomas that are a major cause of mortality in NF1 patients; this often occurs via an intermediate tumor type known as an atypical neurofibroma [8, 9].

The *NF1* gene encodes a protein called neurofibromin, which functions as a key negative regulator of rat sarcoma virus (RAS). Neurofibromin contains a central GTPase-activating protein (GAP)-related domain that accelerates the hydrolysis of active RAS-GTP to its inactive GDP-bound form, thereby constraining RAS signaling [10–12]. *NF1* gene mutations compromise the function of neurofibromin, leading to increased activation of RAS and its downstream effector pathways, including the RAF-MEK-ERK (MAPK) cascade [13–15]. Sustained activation of the MAPK pathway is believed to be a central driver of cellular proliferation and tumorigenesis in NF1. Indeed, the MEK inhibitors (MEKi) selumetinib, trametinib, and mirdametinib have been investigated in clinical trials [16, 17], and MEK inhibition is FDA-approved for inoperable PNs. Both selumetinib and mirdametinib cause significant tumor volume reduction and improve patient-reported outcomes in most children and adults [18, 19]. However, not all patients respond, and the tumor shows regrowth if treatment is ceased due to the side effects. Therefore, alternative strategies or novel therapeutic targets for PN are needed. It may also provide new insights and feasibility for combination therapy to overcome the limitations of MEK inhibitor monotherapy.

Here, we focus on the contribution of protein phosphatases in counteracting the hyperactivation of oncogenic kinases in NF1 as a possible alternative to or combination strategy with MEK inhibition. Protein phosphatases are classified into several families, including the Phosphoprotein Phosphatases (PPP) family (PP1, PP2A, PP2B, PP4, PP5, PP6), the Metal-dependent Protein Phosphatases (PPM) family (PP2C), the protein tyrosine phosphatases (PTPs) family, and the dual-specificity phosphatases (DSPs) family [20]. PP2A is a member of the PPP family, a group of enzymes that typically exist as multi-subunit complexes consisting of a catalytic subunit and one or more regulatory subunits, and are responsible for more than 90% of serine/threonine dephosphorylation events [21]. The PP2A holoenzyme is a heterotrimeric complex composed of a scaffold subunit A, a catalytic subunit C, and a regulatory subunit B [22, 23]. The two genes, *PPP2R1A* and *PPP2R1B,* encode two isoforms of subunit A. Similar to subunit C, encoded by *PPP2CA* and *PPP2CB*. There are fifteen different genes that give rise to more than twenty distinct isoforms of subunit B. The B subunit confers subcellular location and substrate specification to the PP2A enzyme complex [24]. A detailed list of the genes and their encoding subunits is provided in Supplemental Table 1.

We are mostly interested in PP2A mainly because (1) We previously found mutations in *PPP2R3A,* a gene encoding a B regulatory subunit of PP2A, in 3 out of 9 neurofibromas Schwann cells [25]; (2) Multiple lines of evidence support PP2A as an important tumor suppressor, including the oncogenic roles of its endogenous inhibitors SET and CIP2A [26–28] and the pharmacological inhibitor okadaic acid (OA) in various cancers and mouse carcinogenesis models [29, 30], as well as its targeting by the small tumor antigen (small-t) of the transforming DNA viruses SV40 and polyomavirus [31, 32]; (3) Through its association with distinct B regulatory subunits, PP2A regulates a wide range of signaling pathways—including RAS/MEK/ERK, mTOR/AKT, Wnt, and Hippo—as well as fundamental cellular processes such as cell cycle control, proliferation, and apoptosis [33].

FTY720 (Fingolimod) has emerged as a potent and selective activator of PP2A in oncological contexts [34–36]. It directly binds to and antagonizes SET, an endogenous inhibitor of PP2A, thereby promoting the reactivation of the PP2A holoenzyme, restoring its tumor-suppressive phosphatase activity [37, 38]. Beyond its role as an activator of phosphatase activity, when FTY70 is phosphorylated by sphingosine kinase 2 (SphK2), it becomes FTY720-phosphate [39]. FTY720-phosphate induces receptor internalization and degradation of sphingosine-1-phosphate receptor 1 (S1P₁). Thus, FTY720 acts as a functional S1P_1_ antagonist, preventing lymphocyte egress from lymphoid organs and sequestering T-cells in lymph nodes [40, 41]. T cells are required for neurofibroma development in the *DhhCre; Nf1fl/fl* mouse model [42]. Whether FTY720 plays roles in neurofibroma, and if so, whether its effects are on Schwann cells and/or T cells, remains unknown.

Here, we show that PP2A subunits and activity are decreased in human neurofibroma tissues and neurofibroma Schwann cells and could be restored by FTY720. FTY720, alone or in combination with MEKi, reduced the tumor sphere formation by Schwann cell progenitors dissociated from human and mouse neurofibromas, inhibited Schwann cell proliferation, and induced Schwann cell apoptosis, in a partially PP2A phosphatase activity-dependent manner. In vivo, studies using an *Nf1* genetically engineered mouse model showed that FTY720 single agent and combination treatment reduce tumor burden. We suggest that FTY720 is a promising candidate for NF1 treatment.

## Methods and materials

### Mouse husbandry

In vivo mouse experiments were conducted at two independent institutions: Xuzhou Medical University (XZMU, Xuzhou, China) and Cincinnati Children's Hospital Medical Center (CCHMC, Cincinnati, OH, USA). All animal procedures were approved by the Institutional Animal Care and Use Committees (IACUC) at each institution. All mice were housed in temperature- and humidity-controlled facilities on a 12-h dark/light cycle with free access to food and water. The experiments shown in Fig. 3 were conducted at XZMU using *DhhCre* and *Nf1* flox/flox (*Nf1fl/fl*) mice purchased from Cyagen Bioscience Inc. of China. *DhhCre* mice were on an FVBN strain background. *Nf1fl/fl* mice were on a background of C57BL/6. The experiments shown in Fig. 5 were conducted at CCHMC using *DhhCre; Nf1fl/fl* mice on a largely C57BL/6 background, with some residual SV129 and FVBN. In both experiments, the *DhhCre; Nf1fl/* + mice are maintained as male breeders, and *Nf1fl/fl* mice are maintained as female breeders to generate the required genotype (*DhhCre; Nf1fl/fl*). Mouse genotyping uses primers as follows:

floxF1: AGGTGCATAGTACATGTTTTGGTC.

floxR1: TTAGAAACTTGCAGTTCCCTGTGAG.

floxF2: CTGGGGAATCACTTGGTCTGTAA.

floxR2: TCATCCCACTGAGCACCATTTTA.

CreF1: GTCTATCAGTAGTAGGTTCCAGGTTCC.

CreR1: GAAGCATTTTCCAGGTATGCTCAG.

### Selumetinib and FTY720 Dosing (longer duration for restoring PP2A)

*DhhCre; Nf1fl/fl* (4-month-old) mice were stratified into four groups, each with age- and sex-matched animals to minimize the potential non-pharmacological influences. The four groups were administered the following treatments: (1) vehicle control; (2) the MEK inhibitor selumetinib (AZD6244, HY50706, MedChemExpress); (3) FTY720 (HY-12005, MedChemExpress); and (4) a combination of the above two reagents. The vehicle was 10% DMSO, 40% PEG-300, and 5% Tween-80 in 0.9% NaCl. For the single-drug reagent, Selumetinib or FTY720 was dissolved in DMSO (10% of total delivery volume) at a dose of 25 mg/kg and 5 mg/kg, respectively, and then added to 40% PEG-300, 5% Tween-80 in sterile saline. For the combination treatment, both drugs were co-solubilized in DMSO at their respective single-agent doses (25 mg/kg selumetinib and 5 mg/kg FTY720). They were then added to 40% PEG-300 and 5% Tween-80 in sterile saline. Each treatment was administered at a total volume of 200 µl following a schedule of 5 consecutive days of treatment per week with 2 days of rest. All groups received their assigned treatments for a total duration of 8 weeks. In accordance with animal research guidelines, any mouse exhibiting sustained body weight loss exceeding 20% of initial weight was humanely euthanized.

### FTY720 dosing (suppressing T cells)

Mice were administered FTY720 (10 mg/kg body weight) or vehicle control (1% DMSO, 40% PEG300, 5% Tween-80 in sterile saline) once daily by oral gavage for 30 consecutive days. The dosing volume was adjusted according to individual body weight to ensure accurate delivery. FTY720 was freshly prepared each day in the appropriate vehicle immediately before administration. All animals were monitored daily for general health, body weight, and signs of distress throughout the treatment period. At the conclusion of the treatment, spleen, lymph node, and peripheral blood samples were collected 2 h after the final dose for flow cytometric analysis. In addition, tumor tissue, associated nerves, and dorsal root ganglia (DRG) were harvested for immunohistochemical evaluation.

### Flow cytometry

Flow cytometric analysis of immune cell populations in spleen, lymph node, and peripheral blood was performed as previously described [42]. Briefly, single-cell suspensions were prepared, stained with fluorochrome-conjugated monoclonal antibodies against lineage-specific markers, and analyzed on a Cytek Aurora spectral flow cytometer (Cytek Biosciences) using SpectroFlo software. Data were compensated and unmixed using single-stained controls, and gating strategies were established using fluorescence-minus-one (FMO) controls. Dead cells and doublets were excluded based on forward and side scatter parameters and viability dye staining. Data were analyzed using FlowJo software, and identical gating strategies were applied across all samples.

### Immunohistochemistry

Following euthanasia, tumor tissues, associated peripheral nerves, and dorsal root ganglia (DRG) were carefully dissected and immediately fixed in 4% paraformaldehyde at 4 °C for 24 h. Samples were then cryoprotected in 30% sucrose, embedded in optimal cutting temperature (OCT) compound, and sectioned at 10–20 µm thickness using a cryostat. Tissue sections were permeabilized with 0.3% Triton X-100 in PBS and blocked with 5% normal serum for 1 h at room temperature. Sections were incubated overnight at 4 °C with primary antibodies against CD3, followed by fluorophore-conjugated secondary antibodies for 1 h at room temperature. Nuclei were counterstained with DAPI. Slides were mounted using antifade medium and imaged using a confocal laser scanning microscope or fluorescence microscope under identical acquisition settings for all experimental groups. Image analysis and quantification were performed using ImageJ (NIH).

### Mouse dissection and tumor number and size calculation

Two hours after final dose administration, mice were placed into an isofluorane-filled chamber until breathing ceased. Mice were perfused with 0.9% NaCl (Saline) and 4% PFA through cardiac puncture. To quantify tumor numbers and size, the mouse body was decalcified and dissected under a dissecting microscope to obtain the spinal cord with attached DRG and nerve roots. A tumor was defined as a mass surrounding the DRG or nerve roots, with a diameter greater than 1 mm, measured perpendicular to DRG/nerve roots. Tumor numbers within each mouse and the diameter of each tumor were measured with ImageJ.

### Tumor Schwann cell progenitors (SCP) culture and sphere counting

A freshly dissected human plexiform neurofibroma sample was transported on ice to the laboratory within 24 h post-operation. Or a fresh mouse neurofibroma was dissociated from a *Dhhcre;Nf1 fl/fl* mouse (7–9 months age). Fresh tumor tissues were dissociated in dissociation medium [L-15 medium (Yuchun Biology, YC-3029), Pen/Strep/Ampho (Procell system, PB180121), Collagenase type I (Life-ilab, AC15L041), and Dispase II (Sigma-Aldrich, 04942078001)] for 3–4 h at 37 °C in a shaker and obtained single-cell suspensions with narrow-bore pipettes. We then plated the cells in 10 cm Poly-L-Lysine pre-coated plates, culturing with primary SC medium [10%FBS + 2 µM forskolin (Apexbio) and 10 ng/ml Neuregulin-1-Beta (Peprotech)] to recruit the Schwann cell progenitors (SCP). We washed cells with 1xPBS 2–3 times within 24 h before re-plating cells. Then, cells were dissociated with 0.05% trypsin (Vicmed) and re-plated in 24-well low attachment plates (Corning). The free-floating cells were cultured and formed spheres in a serum-free medium with EGF and FGF, N2, B27, and Heparin as previously reported [43]. For passage, we dissociated spheres with 0.05% Trypsin (Vicmed) at 37 °C for 3–5 min. For drug treatment and sphere counts, we plated SCP cells at low density (250/cm2) to avoid sphere fusion (500 cells/well in 24-well plates). Cells were treated with the following agents: solvent control (DMSO), AZD-6244 (5 μM) alone, FTY720 (2 μM) alone, or a combination of AZD-6244 (5 μM) and FTY720 (2 μM) for human SCP cells or different dosage of FTY720 and/or OA as indicated in the Figure for mouse SCP cells. The plates were imaged using an inverted microscope at 12-h intervals from Day 1 to Day 4 after treatment. The number of tumor spheres was counted and analyzed with GraphPad Prism.

### Human gene expression data

We analyzed gene expression levels in human (GEO accession: GSE14038, human) microarray data. The heatmaps represent log2 fold change to the average expression levels of 10 normal cultured human SC samples (NHSC) or to normal human nerves.

### Human tissue collection

A freshly dissected human plexiform neurofibroma sample or a part of nerve was transported on ice to the laboratory within 24 h post-operation. All procedures were performed with patient and physician consent in accordance with national ethical guidelines for human research samples at Department of Plastic and Reconstructive Surgery, Shanghai Ninth People’s Hospital, Shanghai, China. Fresh tissues were either dissociated in dissociation medium or rapidly frozen in liquid nitrogen and stored at -80 for subsequent analysis by Western blotting and PP2A phosphatase activity assays.

### Primary human Schwann cell (SC) culture

The primary human neurofibroma Schwann cells were dissociated and obtained from the neurofibroma tissue of human patients as described above. The cells were constitutively cultured in DMEM with 10% FBS, 1% Penicillin/Streptomycin, 2 µM forskolin (Calbiochem) and 10 ng/ml Neuregulin-1-Beta (Peprotech) adherently in 10 cm poly-L-Lysine pre-coated plates. 0.05% Trypsin (Life Technology) was used for passage.

### PP2A phosphatase activity assay

PP2A phosphatase activity was examined in plexiform neurofibroma tumor tissues and primary cells using the Ser/Thr Phosphatase Assay System (Promega, V2460) following the manufacturer's instructions. Briefly, the Phosphate Standards provided by the system were diluted in a gradient to generate a standard curve. For the preparation of tumor extracts or cell lysates, neurofibroma tissue samples (~ 1 cm^3^) or pellets of cultured primary Schwann cells (~ 1 × 10⁶ cells) were homogenized or lysed in 3 mL of phosphatase storage buffer. The lysates were then subjected to centrifugation using the provided Spin Columns to remove endogenous free phosphate. Subsequently, 10 μL of the column-eluted fractions were transferred to a standard 96-well plate and mixed with 10 μL of 5 × PP2A reaction buffer and phosphopeptide substrate (1 mM), bringing the final reaction volume to 50 μL. The mixture was incubated at 37 °C for 1 h to allow dephosphorylation. The reaction was terminated, and color development was initiated by adding 50 μL of a molybdate dye/additive mixture, followed by incubation at room temperature for 30 min. Absorbance was measured at 600 nm using a microplate reader. To compare PP2A activity between normal nerve tissue and neurofibroma tumors, equal amounts of total protein extract from each tissue type were loaded into individual wells of a 96-well plate. Each sample was analyzed in three technical replicates. The same experimental procedure was applied to cells treated with DMSO, FTY720 or a combination of FTY720 and OA.

### Cell growth curve assay

Cell growth curves were assessed using the CCK-8 (Cell Counting Kit-8) assay and the MTT (3-(4,5-dimethylthiazol-2-yl)-2,5-diphenyltetrazolium bromide) assay following treatment with various compounds. Briefly, cells were seeded in 96-well plates at a density of 1,500 cells per well in 100μL of medium, with three replicates per condition. Upon attachment, cells were treated with DMSO, Selumetinib (AZD-6244, 8 µM), FTY720 (5 µM), or the combination of Selumetinib (8 µM) and FTY720 (5 µM), followed by a CCK8 assay. Or cells were treated with DMSO, FTY720 (5 µM), Okadaic Acid (1 nM), or the combination of Okadaic Acid (1 nM) and FTY720 (5 µM), following with MTT assay. At different time points after treatment, for the CCK-8 assay, 10 μL of CCK-8 reagent was added to each well, followed by incubation at 37 °C for 2 h in the dark. Absorbance was measured at 450 nm using a microplate reader. For the MTT assay, 60 µL of MTT solution (MTT: MEM 1:50 v/v) was added to each well and incubated for 2 h at 37 °C. The resulting formazan crystals were dissolved in 100 µL of Methanol, shaking for 10 min. Absorbance was measured at 595 nm using a microplate reader.

### Cell apoptosis assay

Cell apoptosis was evaluated using an Annexin V-FITC/PI Apoptosis Detection Kit (KGI Bio, KGA1102-100). After 48 h of treatment with DMSO (solvent control), Selumetinib (AZD-6244, 8 µM), FTY720 (5 µM), Okadaic Acid (1 nM), AZD-6244&FTY720 combination, or Okadaic Acid (1 nM) and FTY720 (5 µM) combination, approximately 1 × 10^5^ cells were collected, washed twice with PBS, and resuspended in 100 μL of 1 × Binding Buffer. Then, 5 μL of Annexin V-FITC and 5 μL of Propidium Iodide (PI) were added to the cell suspension. After gentle mixing, the cells were incubated for 15 min at room temperature in the dark. A 30 μL aliquot of the stained cell suspension was placed on a glass slide and immediately visualized under a fluorescence microscope. Apoptotic cells (Annexin V + /PI- and AnnexinV + /PI +) were imaged and quantified using Image J for further analysis.

### Wound healing assay

Primary human Schwann cells isolated from plexiform neurofibromas were plated onto poly-L-lysine (Solarbio)-coated 6-well plates at a density of 6 × 10^5^ cells per well. After 24 h of incubation to allow cell adhesion, a linear scratch wound was generated in the confluent monolayer using a sterile 200 μL pipette tip. The cells were gently washed with PBS to remove debris and dislodged cells. Subsequently, the cells were cultured with medium with either DMSO (solvent control), Selumetinib (AZD-6244, 5 μM), FTY720 (2 μM), Okadaic Acid (1 nM), a combination of Selumetinib (5 μM) and FTY720 (2 μM), or a combination of Okadaic Acid (1 nM) and FTY720 (2 µM) for 24 h. Images of the wound area were acquired at 0,6,12, and 24 h post-scratching. The extent of cell migration was quantified by measuring the wound area using ImageJ software, and statistical analyses were performed with GraphPad Prism.

### qRT-PCR

Primers for testing the mRNA level of *PPP2R1A* and *PPP2CA* are:

2R1A qF: AATGAGGACGTTCAGCTTCGC.

2R1A qR: GTGAAGGTTCCCAGCTGTTCT.

2CA qF: GGCATCATGGACGAGAAGGTG.

2CA qR: CATCGAACCTCTTGCACGTTG.

### Western blot

Antibody information for Western blot experiments is *PPP2R1A* polyclonal antibody (Proteintech, 15882-1-AP); PP2CA polyclonal antibody (Proteintech, 13482-1-AP); Actin (Proteintech, 11313-2-AP); phospho-ERK monoclonal antibody (Proteintech, 80031-1-RR); ERK1/2 polyclonal antibody (Proteintech 11257-1-AP); Neurofibromin Recombinant Monoclonal antibody (Abcam, Ab238142); HRP-conjugated Goat Anti-Rabbit IgG(H + L) (Proteintech, SA00001-2).

### Immunofluorescence

Cells were fixed with 4% paraformaldehyde for 15 min at room temperature, permeabilized with 0.1% Triton X-100 in PBS for 10 min, and blocked with 5% BSA in PBS for 30 min. Samples were then incubated with primary antibodies against S100 (15146-1-AP) overnight at 4 °C. After washing with PBS, cells were incubated with fluorophore-conjugated secondary antibodies (Alexa Fluor 594, Invitrogen) for 1 h at room temperature in the dark. Nuclei were counterstained with DAPI (1 µg/mL) for 5 min. Images were acquired using a fluorescence microscope (Olympus).

## Results

### The A and C subunits of PP2A are significantly reduced in neurofibroma

**Fig. 1:**
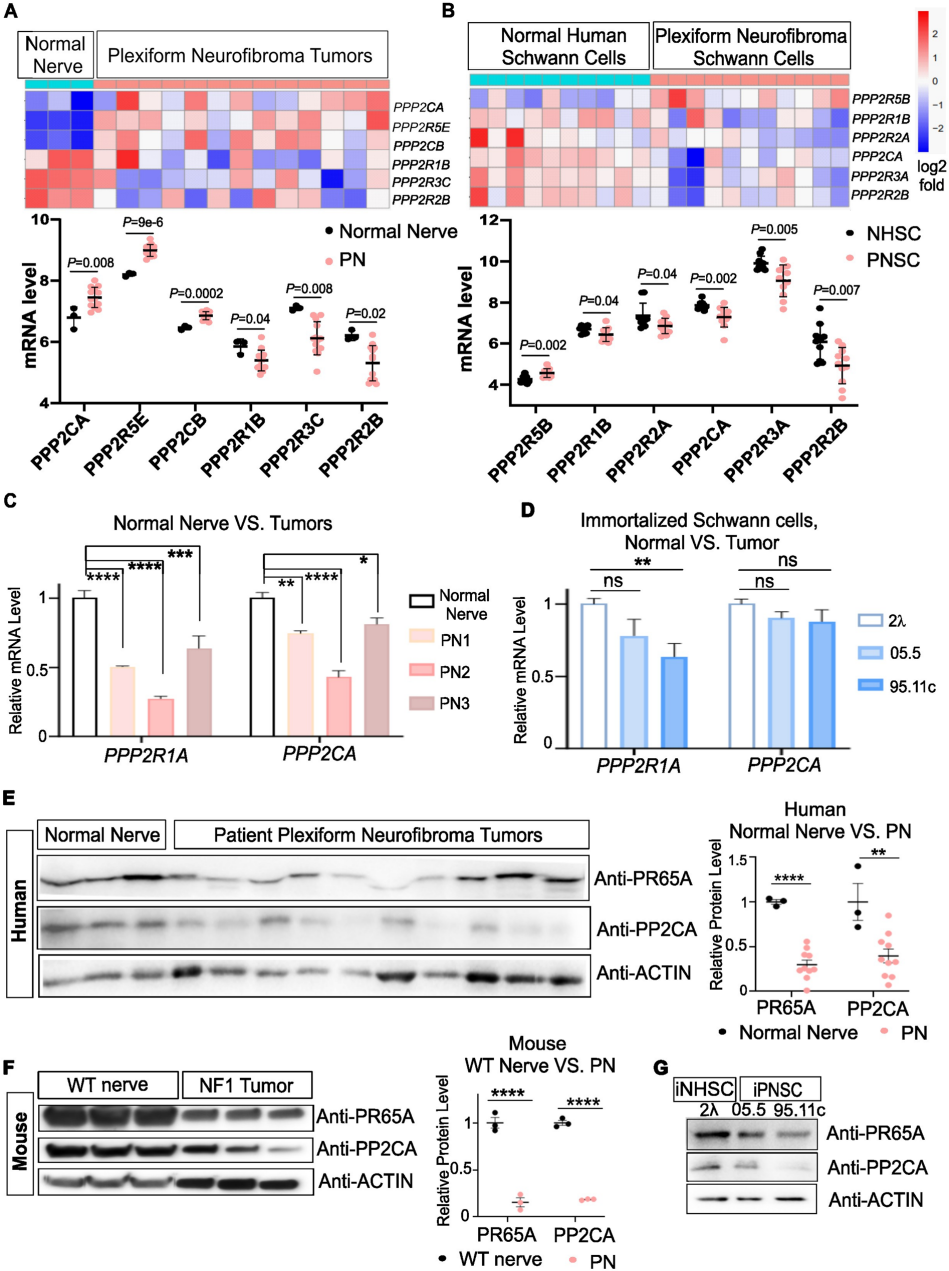
Expression of the A and C subunits of PP2A is significantly decreased in plexiform neurofibromas.** A, B**. Heatmap of DNA microarray showing the subgroup of PP2A subunit genes with dramatically deregulated expression. Quantitative dot plots are placed beneath the corresponding heatmap. NHSC: normal human Schwann cells, PNSC: plexiform neurofibroma Schwann cells. Normal nerve (n = 3), Plexiform neurofibroma tumors (n = 13), NHSC (n = 10), PNSC (n = 11). **C, D** Reduced expression of the gene *PPP2R1A* and *PPP2CA* in neurofibromas and immortalized neurofibroma Schwann cells was confirmed by the results of quantitative real-time PCR (qRT-PCR). One normal nerve control and 3 PNs from different patients were used in C. PN: plexiform neurofibroma. In D, 2λ is an immortalized normal human Schwann cell (iNHSC)**;** 05.5 and *95.11bc* are immortalized Schwann cells derived from plexiform neurofibromas (iPNSC) [44]**. E** Western blot analysis of the A and C subunit proteins in human normal nerve and plexiform neurofibroma tissues. Normal nerves (n = 3 from three otherwise healthy individuals who underwent limb amputation following accidental trauma) and plexiform neurofibromas (n = 10 from different patients) are collected for the experiments. To quantify the Western blot results from human nerve and tumor samples, we used Fiji (ImageJ) software. The signal intensity of each band was normalized to the corresponding actin control. Differences between the control and the experimental groups were analyzed using an independent-samples t-test with Welch's correction for unequal variances. All data were expressed as mean ± SEM. ****p < 0.0001. **F** Results of Western blot of PR65A and PP2CA in mouse normal nerve (n = 3) and plexiform neurofibroma (n = 3) tissues from 6 different mice. Following the quantification using the same method mentioned above.** G** Results of Western blot of PR65A and PPCA in immortalized normal human Schwann cells and immortalized plexiform neurofibroma Schwann cells

To investigate the role of protein phosphatase 2A (PP2A) in type I neurofibroma, we initially analyzed existing DNA microarray data (GEO accession: GSE14038, human). The results revealed differential expression of multiple genes encoding PP2A subunits in neurofibromas compared to normal nerves (Fig. 1A). In neurofibroma Schwann cells, another subgroup of PP2A subunit genes showed dramatic expression changes, including the significantly decreased expression of *PPP2R1B* (encoding the A subunit of PP2A, PR65) and *PPP2CA* (encoding the C subunit of PP2A, PP2CA) (Fig. 1B). Names of the PP2A subunit genes and their encoding proteins are list in supplemental Table 1. We confirmed the reduced expression of subunit A (PR65A) and subunit C (PP2CA) in human neurofibroma tissues using quantitative real-time PCR (Fig. 1C) and Western blot analyses (Fig. 1E). In neurofibromas dissociated from Nf1 mice (*DhhCre; Nf1fl/fl*), PR65A and PP2CA expression were also decreased (Fig. 1F). In the immortalized neurofibroma Schwann cells (iPNSC, 05.5&*95.11bc*), expression of PR65A and PP2CA was also downregulated compared to immortalized normal human Schwann cells (iNHSC, 2λ), albeit to a lesser extent, as assessed by quantitative real-time PCR (Fig. 1D) and Western blot (Fig. 1G). In Supplemental Fig. 1, we presented results from another independent experiment. Neurofibromin was found to be low in human and mouse neurofibroma tissues as well as in iPNSC (05.5 and *95.11bc*), confirming the reliability of our experimental system (Supple. Figure 1A–C). Consistent with our previous findings, the PR65A and PP2CA were also reduced in tumor tissues or iPNSC (05.5 and *95.11bc*). Furthermore, we confirmed the S100 positivity in primary Schwann cells isolated from human neurofibroma tissues, indicating their Schwann cell lineage identity (Supple.Fig. 1D).

### FTY720 rescues the reduced enzymatic activity of PP2A in neurofibroma and neurofibroma-derived Schwann cells

**Fig. 2:**
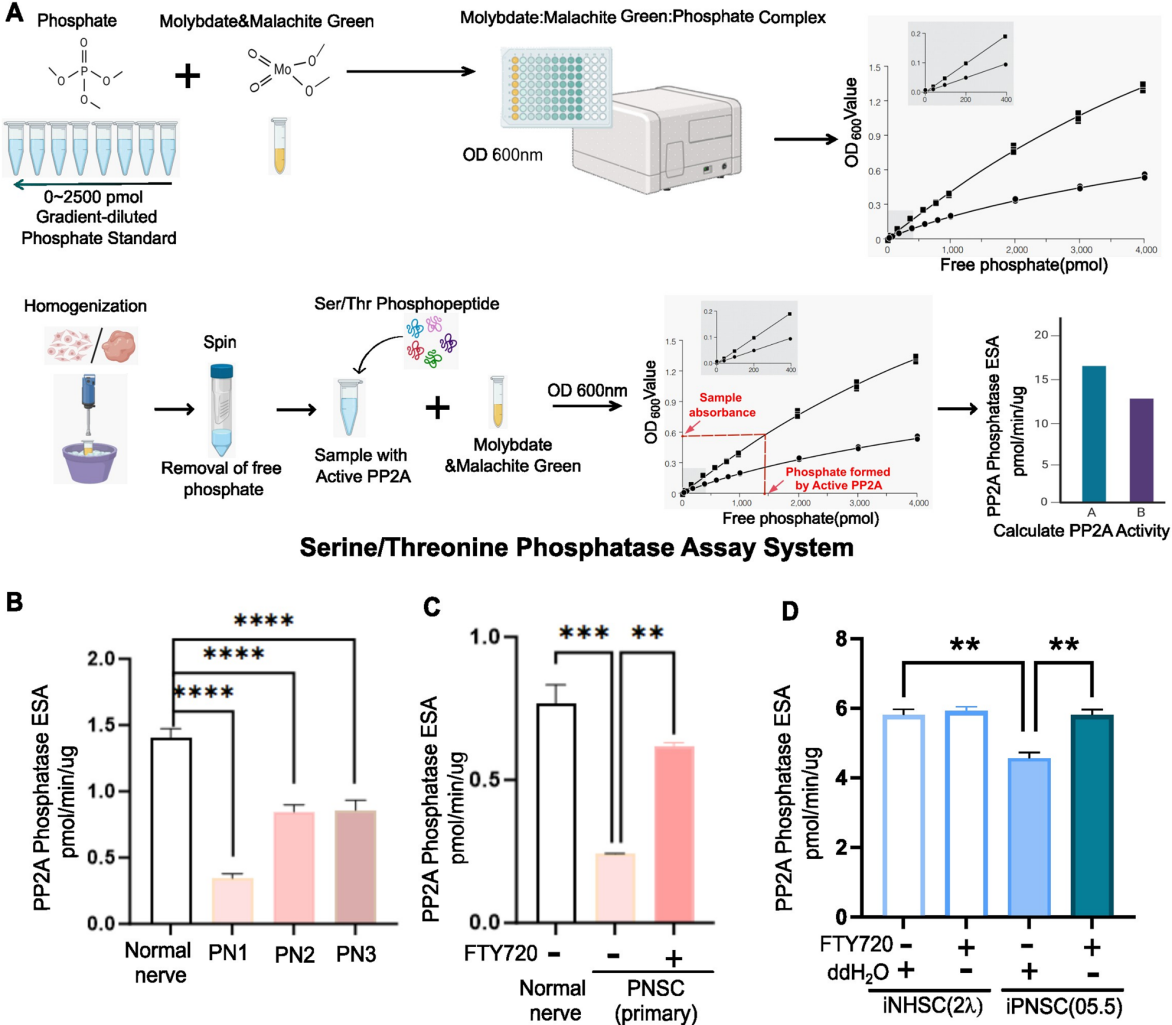
FTY720 rescues the reduced enzymatic activity of PP2A in neurofibroma and neurofibroma-derived Schwann cells.** A** Schematic diagram illustrating the principle and experimental procedure of the PP2A phosphatase activity assay using Ser/Thr Phosphatase Assay System (Promega). **B** Quantitative analysis of PP2A phosphatase activity in normal human nerve tissues (n = 1) and human plexiform neurofibroma tissues (n = 3). PN: plexiform neurofibroma. PN1, 2, and 3 are from three different patients. Data are presented as mean ± SEM; ****p < 0.0001 (by One-way ANOVA). **C** PP2A phosphatase activity in normal nerve (n = 1) and primary Schwann cells isolated from human plexiform neurofibroma (n = 1). Treatment with the PP2A activator FTY720 rescues the impaired phosphatase activity. Data are presented as mean ± SEM; **p < 0.01, ***p < 0.001 (by One-way ANOVA). **D** PP2A phosphatase activity in iNHSC (2λ) and iPHSC (05.5). The suppressed activity of PP2A in iPNSC (05.5) is restored upon treatment with FTY720. Data are presented as mean ± SEM; **p < 0.01 (by Two-way ANOVA)

The structural A subunit and the catalytic C subunit are the core dimeric subunits of PP2A complex. A significant decrease in expression of these subunits is anticipated to compromise PP2A phosphatase activity. To test if this occurs in neurofibroma cells or tissue, we used a Ser/Thr phosphatase assay system, which detects phosphatase activity by measuring the amount of free phosphate removed from the substrate by PP2A phosphatase in crude protein extracts of tumor tissues. Figure 2A shows a schematic illustration of the experimental process. The results demonstrated that PP2A phosphatase activity was significantly reduced in multiple human plexiform neurofibroma lysate (PNs) compared to normal human nerve lysate (Fig. 2B). A significant decrease in PP2A activity was also observed in immortalized human plexiform neurofibromas-derived Schwann cells (iPNSC 05.5) relative to immortalized normal human Schwann cells (iNHSC 2λ) (Fig. 2D). We then isolated and cultured primary Schwann cells from human plexiform neurofibromas (PNSC). PP2A phosphatase activity assays revealed that FTY720 treatment restored PP2A activity in PNSC to a level comparable to that in normal nerve (Fig. 2C). Similar results were obtained in iNHSC (2λ) and iPNSC (05.5) (Fig. 2D). Thus, PP2A enzymatic activity is significantly reduced in neurofibromas and neurofibroma-derived Schwann cells, and FTY720 rescued the low PP2A activity. We then detected whether rescuing PR65A and/or PP2CA expression could restore the PP2A activity. We used lentivirus-packaged *PPP2R1A* and *PPP2CA* overexpression plasmids to infect the iPNSC (05.5), which exhibit reduced PP2A phosphatase activity. Our results showed successful overexpression of PR65A, whereas PP2CA overexpression was not achieved (Suppl. Figure 2A), suggesting that additional regulatory mechanisms may suppress the restoration of PP2CA expression. PR65A overexpression led to a variable change in PP2A phosphatase activity, indicated by three repeats, but not a stable rescue (Suppl. Figure 2B). Unfortunately, our attempt to co-overexpress both of the subunits was unsuccessful, as the double-virus-infected cells exhibited poor viability. We also treated iPNSC (05.5) cells with a pharmacological inhibitor of PP2A—Okadaic Acid (OA), which effectively reversed the rescued PP2A phosphatase activity by FTY720 treatment (Suppl. Figure 2B).

### FTY720 treatment alone or in combination with MEKi reduces the tumor sphere formation and the number of neurofibromas in an NF1 murine model

**Fig. 3:**
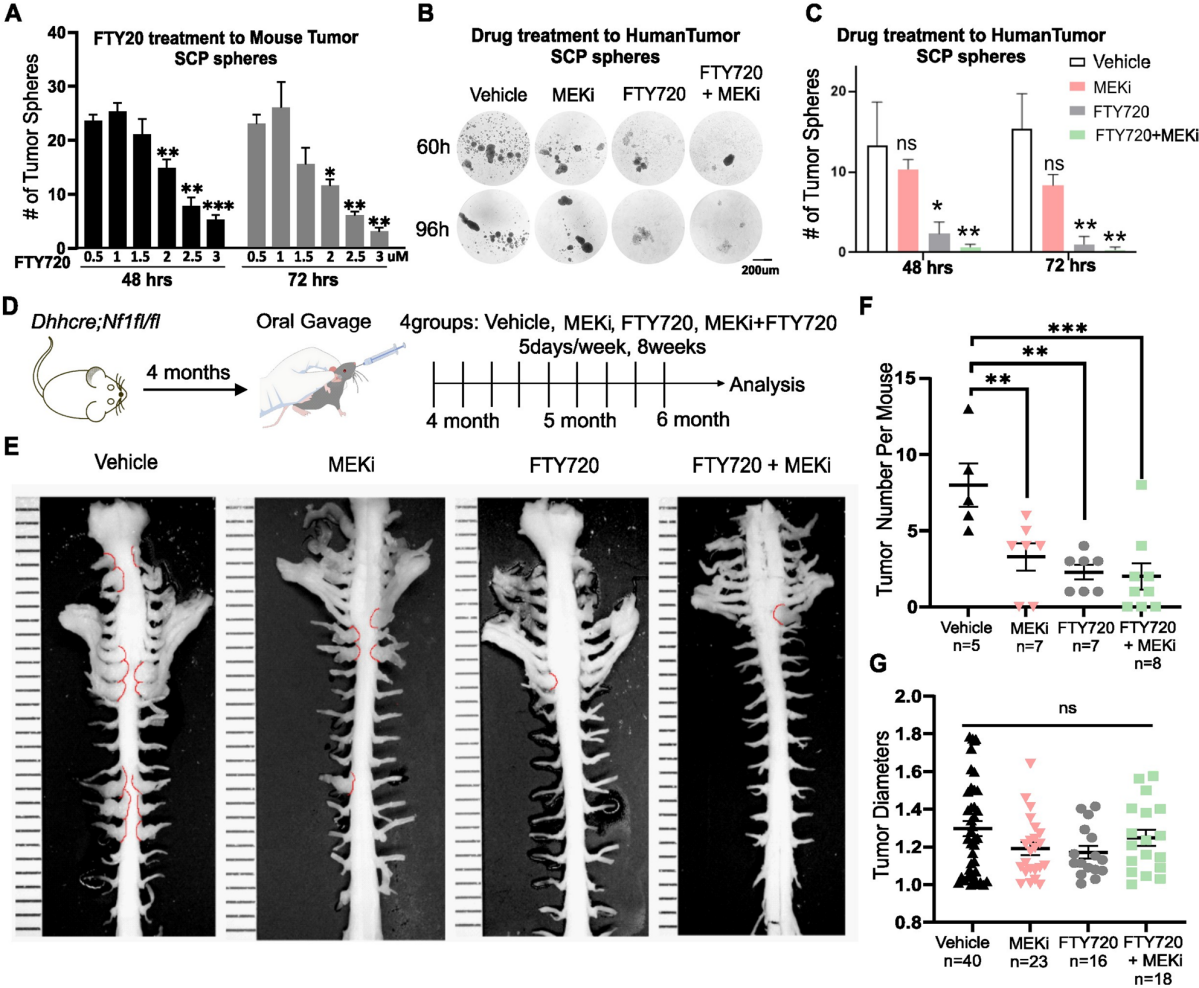
FTY720 treatment alone or in combination with MEKi reduces the tumor sphere formation and the number of neurofibromas in *Dhhcre; Nf1fl/fl* mice. **A** Quantification of neurofibroma tumor spheres formed by mouse neurofibroma Schwann cell progenitor cells treated with the indicated doses of FTY720. **B** Representative images of tumor spheres formed by human neurofibroma Schwann cell progenitor cells following treatment with vehicle, MEKi, FTY720, or the combination of FTY720 and MEKi. **C** Quantification of tumor spheres formed by human neurofibroma Schwann cell progenitor cells treated with vehicle, MEKi, FTY720, or FTY720 plus MEKi.** D** Schematic diagram of the oral gavage administration procedure to the *DhhCre; Nf1fl/fl* mouse. Briefly, mice were dosed from 4 months age. Selumetinib or FTY720 was given at a dose of 25 mg/kg and 5 mg/kg, respectively. For the combination treatment, both drugs were given at their respective single-agent doses (25 mg/kg selumetinib and 5 mg/kg FTY720). Each treatment was administered at a total volume of 200 µl solution following a schedule of 5 consecutive days of treatment per week with 2 days of rest. **E** Representative dissection images of neurofibromas from mice treated with vehicle, MEKi, FTY720, or the combination of FTY720 and MEKi. Red lines mark the compression interface of the spinal cord by the tumor. **F** Quantification of neurofibroma number in mice following treatment with vehicle (n = 5), MEKi (n = 7), FTY720 (n = 7), or the combination (n = 8).** G** Quantification of neurofibroma diameter in mice after treatment with vehicle (40 total tumors from 5 mice), MEKi (23 total tumors from 5 mice, 2 mice bared 0 tumors), FTY720 (16 total tumors from 7 mice), or the combination (18 total tumors from 5 mice, 3 mice bared 0 tumors)

Given that FTY720 can restore PP2A phosphatase activity to *NF1* mutant cells, we decided to test its potential effects on the growth of neurofibroma tumors. To this end, we first treated mouse Schwann cell progenitors (SCP) isolated from neurofibromas with FTY720; SCP exhibit stem/progenitor-like properties and form tumor spheres in vitro. Sphere number decreased with increasing doses of FTY720 (Fig. 3A). A similar effect was observed in SCP spheres derived from human plexiform neurofibromas. Representative micrographs of tumor spheres are shown in Fig. 3B. Quantitative analysis confirmed that FTY720 treatment alone significantly inhibited tumor sphere formation compared to the vehicle control. Moreover, combination therapy with FTY720 and a MEK inhibitor (AZD6244, Selumetinib) further suppressed tumor sphere formation (Fig. 3C). We further evaluated the tumor-suppressing effect of FTY720 in the neurofibroma mouse model (*DhhCre; Nf1 fl/fl*) in longer-duration in vivo experiments. Mice were divided into four groups: vehicle, MEKi, FTY720, and combination treatment with both agents. Based on previously published dosing regimens for MEKi (AZD6244), we administered MEKi at 25 mg/kg once daily and FTY720 at 5 mg/kg once daily. For the combination group, each drug was given at the same dose in combination. Treatment was initiated at 4 months of age when neurofibromas began to form, following a schedule of 5 days of doses per week with 2 days of rest, for a total of 8 weeks of treatment (Fig. 3D). The mice tolerated this regimen well, without observable weight loss or other adverse effects. Post-dissection examination showed normal phenotypes in the liver and spleen (Data not shown). Representative gross images of dissected spinal cords with attached nerves and tumors are shown in Fig. 3E. Both MEKi and FTY720 monotherapy, as well as the combination treatment, effectively reduced the number of neurofibromas formed in vivo (Fig. 3F), while the size of neurofibromas was comparable in each treatment group (Fig. 3G).

### FTY720 reduces tumor sphere formation, Schwann cell proliferation, migration of primary Schwann cells, and induces cell apoptosis in a partially PP2A-dependent manner

**Fig. 4:**
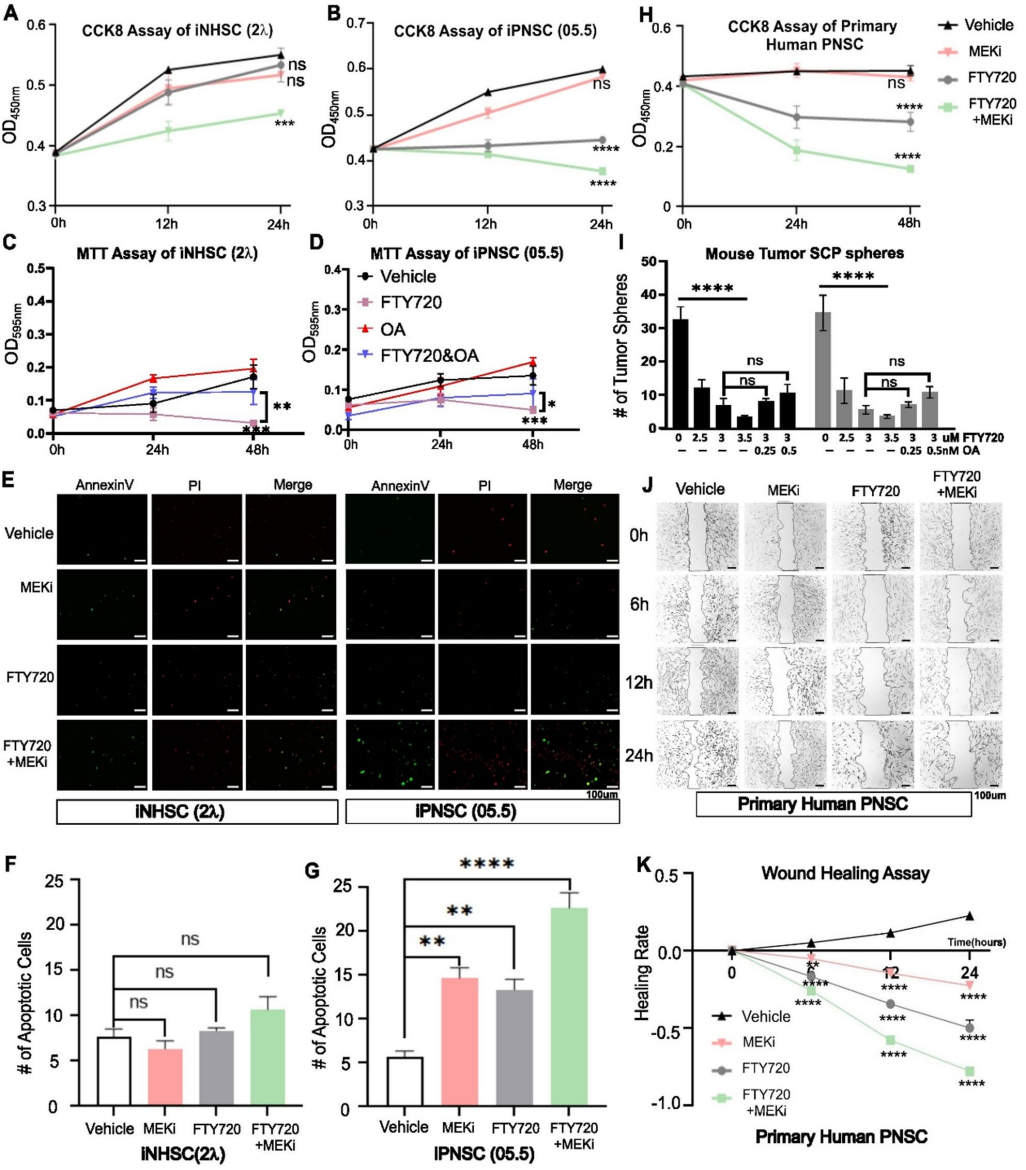
FTY720 inhibits neurofibroma tumor sphere formation, suppresses cell growth and migration of primary and iPNSC, and induces iPNSC apoptosis in a PP2A partially dependent manner. **A **and** B** CCK-8 assay assessing the growth of iNHSC (2λ) and iPNSC (05.5) treated with vehicle, MEKi, FTY720, or FTY720 combined with MEKi. **C **and** D** MTT assay assessing the growth of iNHSC (2λ) and iPNSC (05.5) treated with vehicle, FTY720, OA, or FTY720 combined with OA. All experiments in A-D were performed in triplicate with at least three independent repetitions. Data are presented as mean ± SEM. Statistical analysis was performed using two-way ANOVA. A value of *p* < 0.05 was considered statistically significant. **E.** Representative images of apoptotic cells detected by Annexin V/PI staining in iNHSC (2λ) and iPNSC (05.5) after treatment with vehicle, MEKi, FTY720, or the combination therapy. **F **and** G** Quantitative analysis of apoptosis in iNHSC (2λ) and iPNSC (05.5) following treatment with vehicle, MEKi, FTY720, or FTY720 combined with MEKi. Apoptotic cells are AnnexinV + /PI- plus AnnexinV + /PI + cells, and were quantified from ten randomly selected fields per treatment group following Annexin V/PI staining. Data are shown as mean ± SEM. Statistical significance was determined by one-way ANOVA. *p < 0.05, **p < 0.01, ***p < 0.001. **H** CCK-8 assay measuring the growth of primary human neurofibroma-derived Schwann cells (PNSC) treated with vehicle, MEKi, FTY720, or the combination of FTY720 and MEKi. Quantitative analysis was the same as described in A-D.** I** Quantification of neurofibroma tumor spheres formed by mouse neurofibroma Schwann cell progenitor cells treated with the indicated doses of FTY720 with or without OA. The data represent four replicate wells per treatment group. **J** Representative images of wound healing assay showing the migration of PNSC after treatment with vehicle, MEKi, FTY720, or FTY720 plus MEKi. **K** Quantitative analysis of the wound healing assay presented in **J**. Three replicates were quantified for each time point. Statistical significance was determined by Two-way ANOVA. *p < 0.05, **p < 0.01, ***p < 0.001

To further investigate the mechanism by which FTY720 inhibits neurofibroma formation and to determine whether this effect is mediated by the restoration of PP2A phosphatase activity, we performed cellular functional assays, including assessment of cell growth and apoptosis using iNHSC (2λ) and iPNSC (05.5). CCK-8 assays revealed that FTY720 suppressed cell growth in iPNSC (05.5) but not in iNHSC (2λ). The combination of FTY720 and MEKi slowed the growth of iNHSC (2λ), and significantly suppressed iPNSC (05.5) growth (Fig. 4A, B). The inhibition of cell growth by FTY720 in iNHSC (2λ) and iPNSC (05.5) cells was partially reversed by Okadaic Acid (OA), a pharmacological inhibitor of PP2A (Fig. 4C, D). This finding indicates that the anti-growth effect of FTY720 is partially dependent on its enhancement of PP2A phosphatase activity. In iNHSC (2λ), neither single agent nor combination induced apoptosis in Annexin V/FITC apoptosis assays. In contrast, MEKi and FTY720 monotherapies induced apoptosis to some extent in iPNSC (05.5); combination treatment further enhanced apoptotic cell death (Fig. 4E–G). Similar to the cell growth assay, the FTY720-induced apoptosis in iPNSC (05.5) was also reversed by OA, although this effect did not reach statistical significance (Supple. Figure 3B). To determine effects on cells that are not immortalized, human neurofibroma tumor spheres were dissociated and subjected to adherent culture, generating primary Schwann cells. FTY720 alone and FTY720 combined with MEKi significantly inhibited the growth of these primary human neurofibroma Schwann cells (PNSC) (Fig. 4H). We didn’t get enough cells to detect the rescuing effect of OA on the human PNSC growth. However, we saw a slight rescue of OA treatment to the mouse sphere formation by SCP cells (Fig. 4I). These results indicate that FTY720 inhibits neurofibroma tumor and tumor sphere formation partially through PP2A activation, potently also via other mechanisms. We also used a wound healing assay to test if cell migration is affected by drug treatment. Treatment with MEKi or FTY720 alone, as well as their combination, significantly inhibited the wound healing rate at 12 and 24 h (Fig. 4J–K), indicating suppressed cell migration. Consistent with previous observations, OA treatment partially reversed the anti-migratory effect of FTY720 (Suppl. Figure 3C). Notably, the reduced cell migration observed following drug treatment may also result from the concomitant decrease in cell growth and increase in apoptosis.

### FTY720 treatment increased p-ERK in mouse neurofibromas but not in immortalized human Schwann cells

The activation of RAS/MEK/ERK signaling is considered a major driver of cell proliferation and neurofibroma development. We further detected the effect of FTY720 on this signaling pathway. To our surprise, with FTY720 treatment, the phosphorylated ERK was not decreased, but inversely, it was significantly increased in mouse neurofibromas (Suppl.Fig. 3D). In iPNSC (05.5), p-ERK were dramatically higher than those in iNHSC (2λ), consistent with the expected *NF1* loss. However, neither FTY720 treatment alone nor FTY720 in combination with OA led to significant changes in p-ERK levels. The molecular mechanisms by which FTY720 inhibits neurofibroma cells through partially activating PP2A warrant further investigation.

### FTY720 treatment reduces neurofibroma number but does not alter intratumoral T-cell abundance

**Fig. 5:**
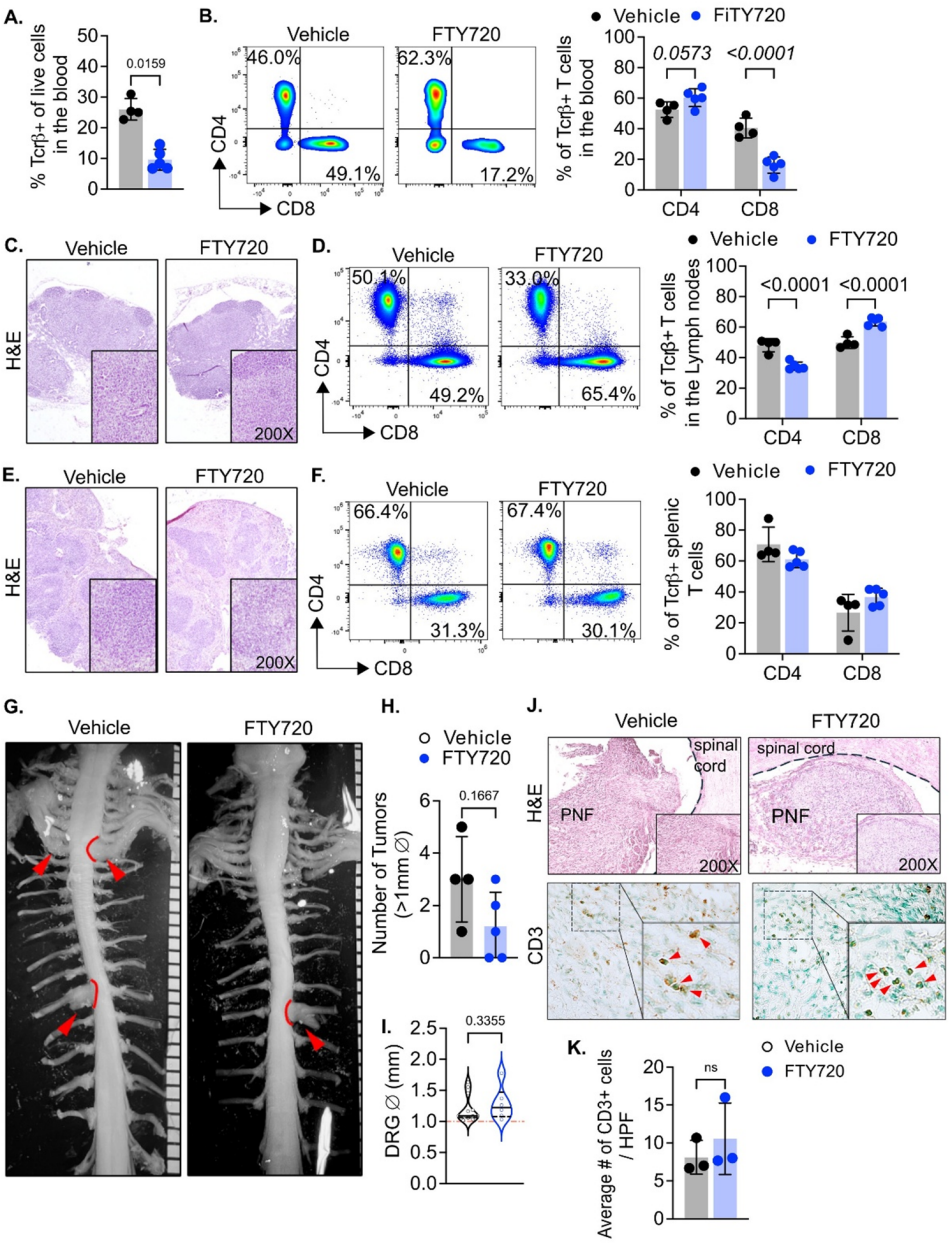
FTY720 treatment alters peripheral and lymphoid T-cell distribution and modulates paraspinal tumor burden in mice. Mice were treated daily with FTY720 (10 mg/kg body weight) or vehicle by oral gavage for 30 days, after which peripheral blood, lymphoid organs, and paraspinal tumors were harvested and analyzed. **A** Representative flow cytometric analysis of circulating TCRβ + T cells. **B** Quantification of the frequency of circulating CD4 + and CD8 + T cells. **C** Representative hematoxylin and eosin (H&E)-stained sections of lymph nodes, with inset showing higher-magnification images (200 ×). **D** Flow cytometric quantification of CD4 + and CD8 + T cells in lymph nodes. **E** Representative H&E-stained spleen sections, with inset showing higher-magnification images (200 ×). **F** Flow cytometric analysis of splenic CD4 + and CD8 + T-cell frequency.** G** Representative gross dissection image of paraspinal tumors. Red arrows indicate tumors, and the red outline denotes spinal cord compression caused by the tumor. **H** and** I **Quantification of tumor number and tumor diameter. **J** Representative tumor sections stained with H&E, with inset showing higher magnification (200 ×), and CD3 immunohistochemistry counterstained with methyl green. Arrows indicate CD3 + T cells. **K** Quantification of CD3 + cells per field. Data are presented as mean ± SD. Statistical analysis was performed as indicated in the Methods

In addition to its action as a phosphatase activator, FTY720 (also known as Fingolimod) is an S1P receptor modulator that blocks lymphocyte egress from lymphoid organs, and trafficking to the site of inflammation [45]. T cells are required for neurofibroma development in the *DhhCre; Nf1fl/fl* mouse model [42].We therefore tested whether FTY720 could alter T-cell abundance in neurofibromas and potentially thereby impact tumor burden. To evaluate the effect of FTY720 on neurofibroma development, we treated *DhhCre; Nf1fl/fl* mice with FTY720 (10 mg/kg/day) daily by oral gavage for 4 weeks. At 2 h following the final dose, spleen, draining lymph nodes, blood, and tumor or DRG/nerve tissues were harvested for immunohistochemistry and flow cytometric analysis of T-cell populations. We first confirmed systemic activity of FTY720 by assessing lymphocyte distribution. As expected, in the blood, FTY720 reduced total T cells (Fig. 5A), driven predominantly by a decrease in CD8 + T cells, whereas CD4 + T cells were not significantly changed (Fig. 5B), consistent with the known mechanism of S1P receptor modulation [46]. In tumor-draining lymph nodes, the overall frequency of T cells was unchanged, but the relative levels of CD4 + T cells decreased, and CD8 + T cells increased in FTY720-treated mice (Fig. 5C, D). Despite these shifts in subset composition, the effector phenotype of CD4 and CD8 + T cells was not significantly altered (Supple. Figure 4). In contrast, no significant changes in T-cell frequency were observed in the spleen (Fig. 5E, F), and spleen size did not differ between treatment and control groups (Supple. Figure 5). We then quantified tumor burden. FTY720-treated mice showed a trend toward a reduction in the total number of neurofibromas compared with vehicle controls, although this difference did not reach statistical significance (p = 0.1). This marginal effect suggests that increasing the sample size might reveal a statistically significant difference. (Fig. 5G–H). However, average tumor size per lesion was not significantly different between groups (Fig. 5I). Notably, the number of CD3 + T cells within tumors was not significantly changed following FTY720 treatment (p = n.s.) (Fig. 5J–K), similar with the number of CD4⁺ and CD8⁺ T cells. FTY720 treatment did not alter the proportion of either subset of T cells in the tumor, neither their activation nor differentiation profiles assessed by CD44 and CD62L expression (Supple. Figure 6). These results indicate that despite systemic sequestration of T cells, FTY720 does not reduce T-cells present in neurofibromas. Together, these data indicate that FTY720 decreases neurofibroma number in the *DhhCre; Nf1fl/fl* model but does not deplete T cells within the tumor microenvironment.

## Discussion

Here, we show that the expression of the PP2A subunits PR65A and PP2CA is significantly downregulated in human and mouse neurofibroma tissues and in Schwann cells, accompanied by a marked reduction in PP2A phosphatase activity. FTY720 restored PP2A activity in both primary and immortalized neurofibroma Schwann cells, significantly inhibiting sphere formation by Schwann cell progenitors and the proliferation of Schwann cells. Importantly, FTY720 combined with the MEK inhibitor selumetinib effectively induced tumor cell apoptosis. In vivo, FTY720 treatment markedly reduced tumor number in an Nf1 genetically engineered mouse model (*DhhCre; Nf1fl/fl*). Together, these findings suggest that PP2A downregulation represents a therapeutic vulnerability in NF1 and that FTY720 is a promising candidate for its treatment.

Notably, in the current study, FTY720 and MEKi monotherapy, or their combination, failed to reproduce the tumor volume reduction previously observed with MEKi treatment. This discrepancy may be attributed to differences in experimental methodologies: (1) In the present study, MEKi was administered only once daily. Given its short metabolic half-life, a single daily dose may not achieve the therapeutic exposure levels previously attained with twice-daily dosing. (2) Due to technical limitations, we were unable to employ MRI to monitor volume changes in the same tumor before and after treatment. The drug’s effect might only manifest as a reduction in tumor number rather than volume shrinkage in different individual subjects. (3) When assessing changes in tumor volume, only tumors larger than 1 mm in diameter were included in the analysis, rather than measuring all dorsal root ganglion nerves. It is possible that a reduction in tumor volume could be detected if data from all dorsal root ganglia were collectively analyzed. Based on what we mentioned above, we cannot ideally distinguish whether FTY720 acts on tumor initiation, early lesion survival, or the regression of established tumors. However, considering the potent pro-apoptotic effects of FTY720 observed in cultured Schwann cells, we speculate that the in vivo reduction in tumor burden may at least in part result from the regression of existing neurofibromas. Further experiments are needed to confirm this.

A limitation of our study is that, while OA reversal suggests PP2A mediates FTY720's effects on proliferation, apoptosis, and migration, we did not identify the downstream signaling pathways involved. PP2A complexes dephosphorylate multiple substrates to regulate multiple cellular pathways. In other settings, phospho-proteomic analysis revealed potential substrates; FTY720 reduced the activity of PP2A-regulated kinases, including ERK1, GSK3β, AURB, and PLK1, and led to suppression of MYC [47]. PP2A inhibits the MAPK pathway by indirectly inhibiting c-Src or directly dephosphorylating ERK [48, 49]. It can inhibit cell survival by dephosphorylating AKT [50–52]. PP2A can also inhibit cell migration by interacting with and regulating RAC [24]. In cervical cancer cells, inhibition of PP2A can stabilize and activate p53 to regulate cell cycle arrest and promote cell apoptosis [24, 53–55]. In the present study, we did not evaluate other PP2A substrates beyond p-ERK.

Interestingly, while FTY720 did not significantly affect p-ERK levels in immortalized Schwann cells, p-ERK was markedly elevated in mouse neurofibromas with FTY720 treatment. This observation argues against a negative regulatory effect of PP2A on ERK but raises the possibility of a positive one, as suggested by some studies [56, 57]. Thus, we speculate that FTY720 may activate PP2A, which in turn positively modulates ERK signaling. This could be mediated by a specific B regulatory subunit, B55α, which negatively regulates inhibitory Raf, thereby relieving Raf inhibition and leading to excessive ERK phosphorylation [56] that neurofibroma cells cannot tolerate. This possibility is reminiscent of a previous study [58], in which inhibition of DUSPs reduced MPNST cell growth and was associated with hyperactivation of both ERK and JNK. How these changes in p-ERK—or potential alterations in other PP2A substrates—contribute to the PP2A-dependent effects of FTY720 in neurofibroma remains to be further investigated.

The observed reduction in PP2A activity and expression in neurofibromas may be related to the PP2A endogenous inhibitor SET (also known as I2PP2A) and/or microRNA-mediated regulation. Notably, SETBP1, a SET-binding protein and stabilizing SET [27, 59], is overexpressed in neurofibromas and malignant peripheral nerve sheath tumors (MPNSTs) [60]. Additionally, miR-155—a microRNA frequently upregulated in neurofibromas and contributing to tumor growth and Schwann cell proliferation[61]—has been shown to repress PP2AC promoter activity, mRNA abundance, and protein levels [62]. Elucidating the regulatory pathways responsible for PR65 downregulation in neurofibromatosis represents a compelling and scientifically important research direction.

Our attempt to overexpress PR65A and/or PP2CA to restore PP2A activity in iPNSC (05.5) was largely unsuccessful. This may be because both A and C subunits are downregulated in neurofibroma, and restoring only one of them is insufficient to rescue overall PP2A activity. The structural complexity of PP2A, derived from its core dimer (A/C) assembling with diverse B subunits into distinct heterotrimeric holoenzymes [23], underlies both the sensitivity of its phosphatase activity to perturbations and the functional complexity of PP2A [63, 64]. As a result, altered expression of a single subunit may be sufficient to modulate global PP2A activity or the activity of a specific signaling pathway regulated by PP2A. As core components of the PP2A catalytic dimer, the expression levels of both A and C subunits are likely tightly regulated, and alterations in either may significantly impact PP2A function [65, 66]. The specific B subunit with which PP2A associates determines its signaling specificity and, in a context-dependent manner, its pro- or anti-tumorigenic function [21]. This context-dependent duality may help explain why Semenova et al. found that the PP2A inhibitor cantharidin is among the most promising candidates for treating MPNST [67], whereas our work suggests that FTY720 suppresses neurofibroma formation, at least in part, by restoring PP2A activity. A key direction for future research is to clarify which specific B subunit‑containing PP2A holoenzyme predominantly regulates tumor development in benign versus malignant settings, and to develop strategies that selectively target this holoenzyme.

Growing evidence highlights the potent anti-tumor properties of FTY720 via activating PP2A. For example, multiple in vitro and in vivo studies demonstrate growth arrest and apoptosis following FTY720 activation of PP2A in cancers, including glioma [68], breast cancer [63], prostate cancer [69], and pancreatic cancer [70]. However, FTY720 has well-established PP2A-independent activities, including S1P receptor modulation [71]. In *DhhCre; Nf1fl/fl* mice, FTY720 treatment resulted in a marked alteration of T cell distribution, characterized by a significant reduction in circulating T cells, predominantly within the CD8 + subset. This observation aligns with FTY720’s mechanism as an S1PR modulator, which sequesters lymphocytes within secondary lymphoid organs by preventing their egress into circulation [72]. The increased presence of CD8 + T cells in the lymphoid tissues suggests enhanced retention or local proliferation contributing to altered immune dynamics. This correlated with a reduction in neurofibroma numbers, although FTY720 therapy did not reduce intratumoral T cell numbers. Overall, this suggests that the reduction in peripheral T cells may dampen systemic inflammation, indirectly affecting tumor growth. T cell expression of the activation markers CD62L and CD44 in neurofibromas and the role of FTY720 in immunomodulation warrant future investigation in this setting.

In neurofibroma clinical trials, nearly 30% of patients treated with MEK inhibitors exhibit poor response, and tumor regrowth is typically observed following dose reduction or drug discontinuation [73]. This is likely because MEK inhibitors as monotherapy have limited capacity to induce apoptosis in neurofibroma cells. Our finding that FTY720 combined with a MEK inhibitor induced apoptosis in immortalized Schwann cells and reduced neurofibroma number suggests that FTY720 may complement this deficiency. Mechanistically, FTY720 may induce apoptosis partially through restoring PP2A activity, and PP2A is known to promote apoptosis by dephosphorylating key survival-related proteins, including AKT, c-MYC, and BCL-2 [52, 74, 75], which sustain tumor cell survival [76, 77]. Additionally, FTY720 may also induce apoptosis through alternative mechanisms, such as altered mitochondrial permeability [78] or sphingosine kinase inhibition, as supported by the partial reversal of its effects by OA and the lack of intratumoral T cell changes in FTY720-treated mice. The precise apoptotic pathways involved—whether via PP2A, mitochondria, SK1, or other effectors—require further investigation. Nevertheless, based on the current evidence, it is reasonable to consider testing the combination of FTY720 and a MEK inhibitor in patients with inoperable plexiform neurofibroma.

However, although we exploited FTY720's immunosuppressive properties to reduce neurofibroma growth—based on evidence that CD8 T cells drive neurofibroma formation [7, 42]—this mechanism also poses clinical risks, including impaired anti-tumor immunity and increased infection susceptibility. Thus, careful risk–benefit assessment and strategies to mitigate systemic immunosuppression are needed. Alternatively, elucidating the detailed downstream pathways by which FTY720 suppresses neurofibromas may enable the development of more selective agents that retain efficacy without compromising systemic immunity. Additionally, our study is limited by the lack of clinical information on the neurofibroma patients from whom neurofibroma samples were obtained, including age, sex, *NF1* mutation status, and pre- or post-operative treatments. Addressing this gap is essential for ensuring the safety of FTY720 in future clinical applications for NF1.

## Supplementary Information

Below is the link to the electronic supplementary material.

Supplementary Material 1.

Supplementary Material 2.

## Data Availability

Human gene expression microarray data was available through GEO accession: GSE14038, human.

## Declarations

### Use of AI

During the preparation of this work the author used Deepseek in order to do the English Revision. After using this tool/service, the author reviewed and edited the content as needed and takes full responsibility for the content of the publication.
