# Supplementary material for: Decreased PP2A expression and activity represent a therapeutic target for plexiform neurofibroma
Source: Acta Neuropathol Commun. 2026 May 11;14:147. doi: 10.1186/s40478-026-02315-w (PMC13366792; doi:10.1186/s40478-026-02315-w)
Supplement: Supplementary file 1 — Supplementary Material 1. [file 40478_2026_2315_MOESM1_ESM.docx]

**Supplementary Data (Original results of WB page)**


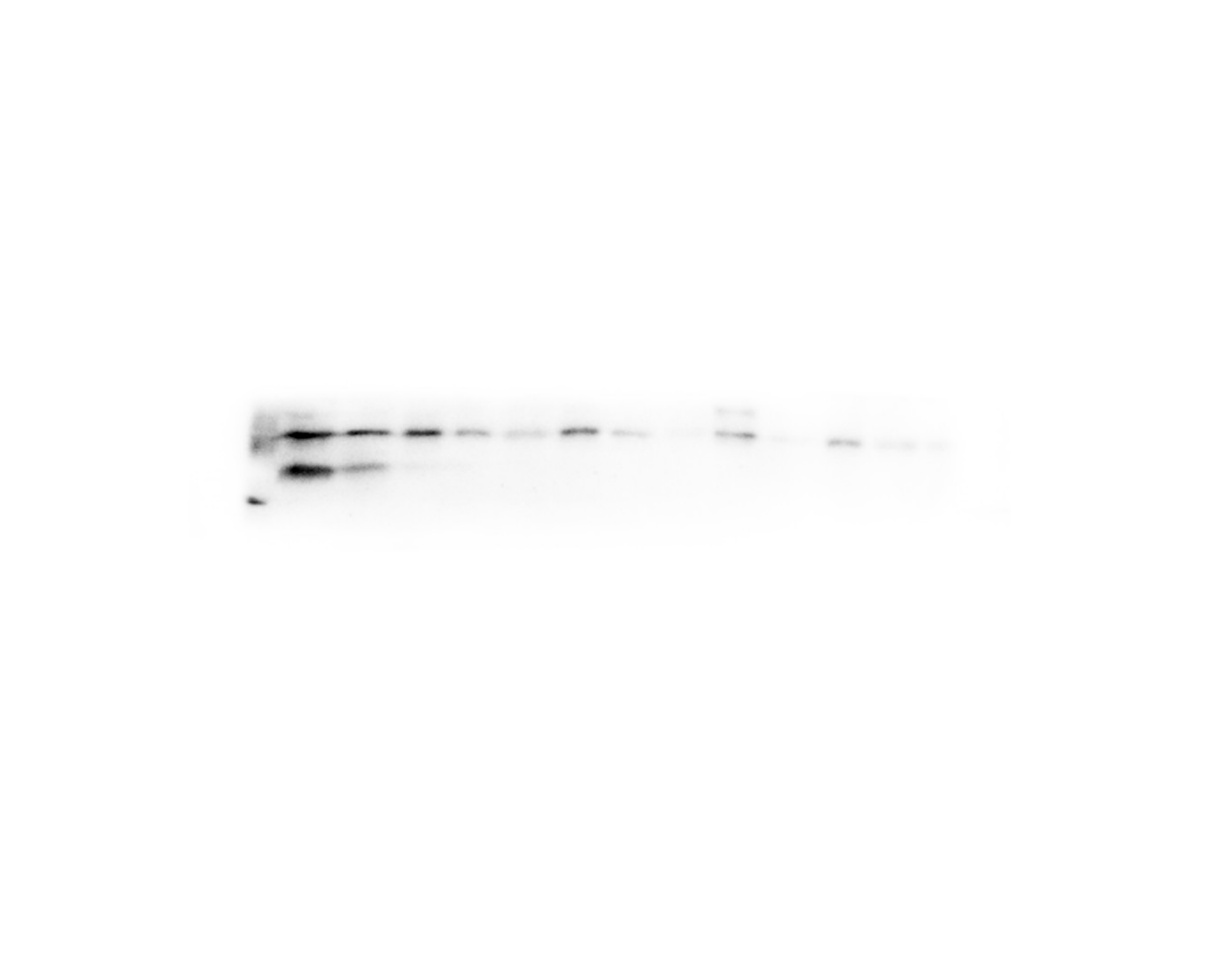

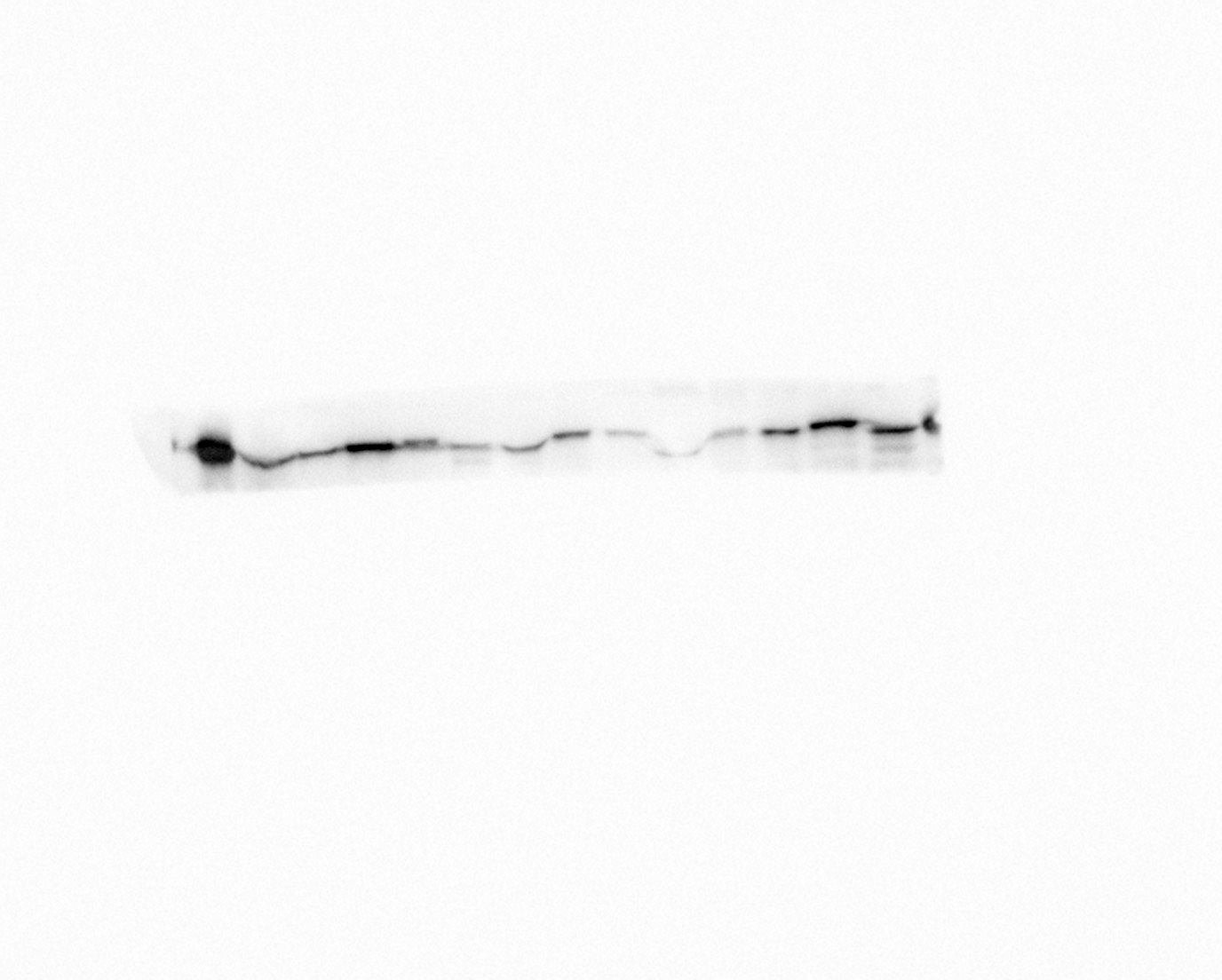
Given the limited availability of precious tumor tissue samples and to avoid the inaccuracies in protein quantification associated with repeated stripping and re-probing of SDS-PAGE membranes (which leads to significant protein loss), we employed a membrane-cutting strategy. Prior to immunoblotting, the membrane was carefully cut into horizontal strips according to the molecular weights of the target proteins, as guided by the pre-stained protein ladder and confirmed by Ponceau S staining. Each membrane strip was then incubated separately with its corresponding primary and secondary antibodies, followed by independent detection.

Normal Nerve

PNFs

**Figure 1 panel E anti-PR65A**

Ladder

70KD

**Figure 1 panel E anti-PP2Ac**

Normal Nerve

PNFs

Ladder

35KD


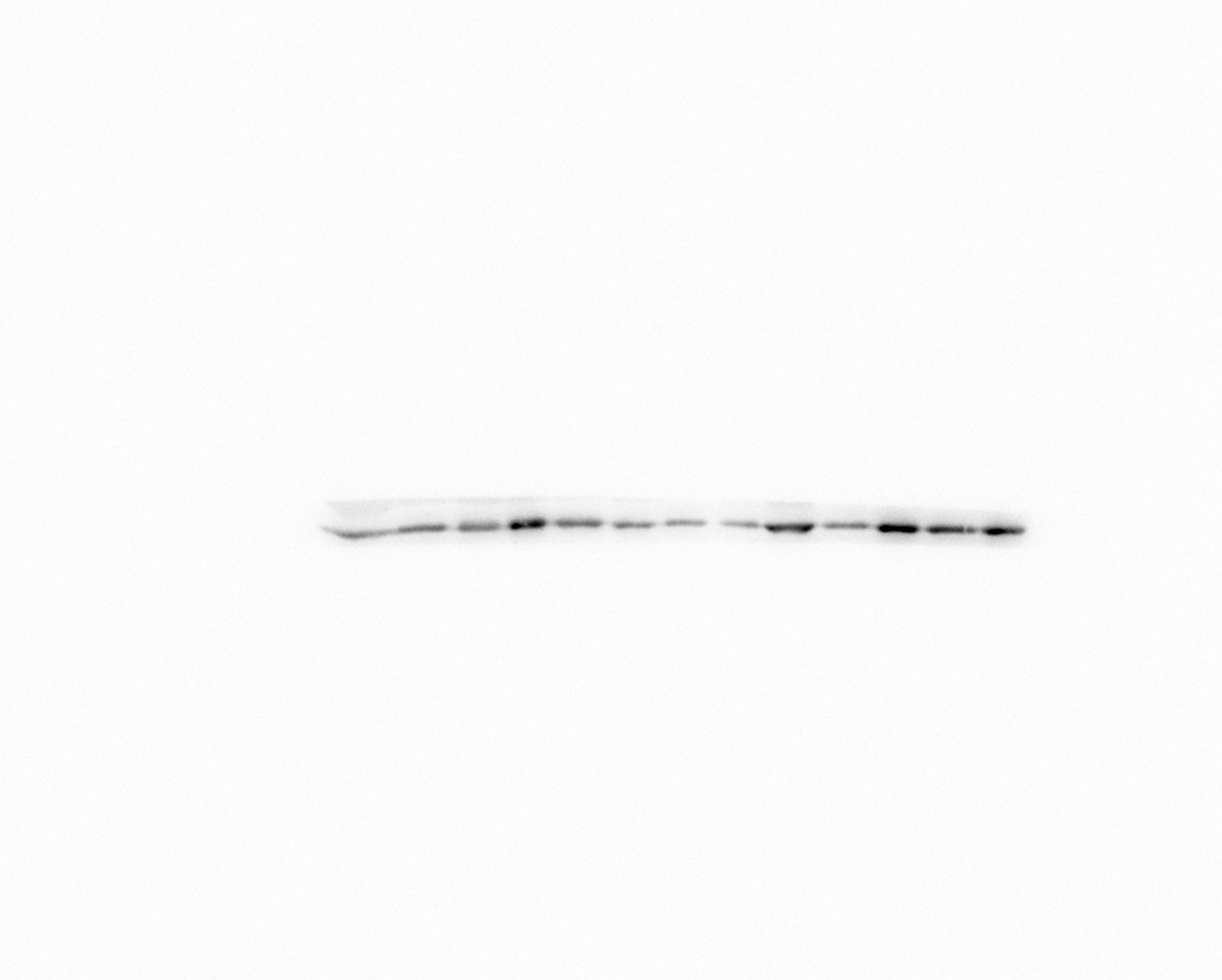


PNFs

Normal Nerve

**Figure 1 panel E anti-Acin**


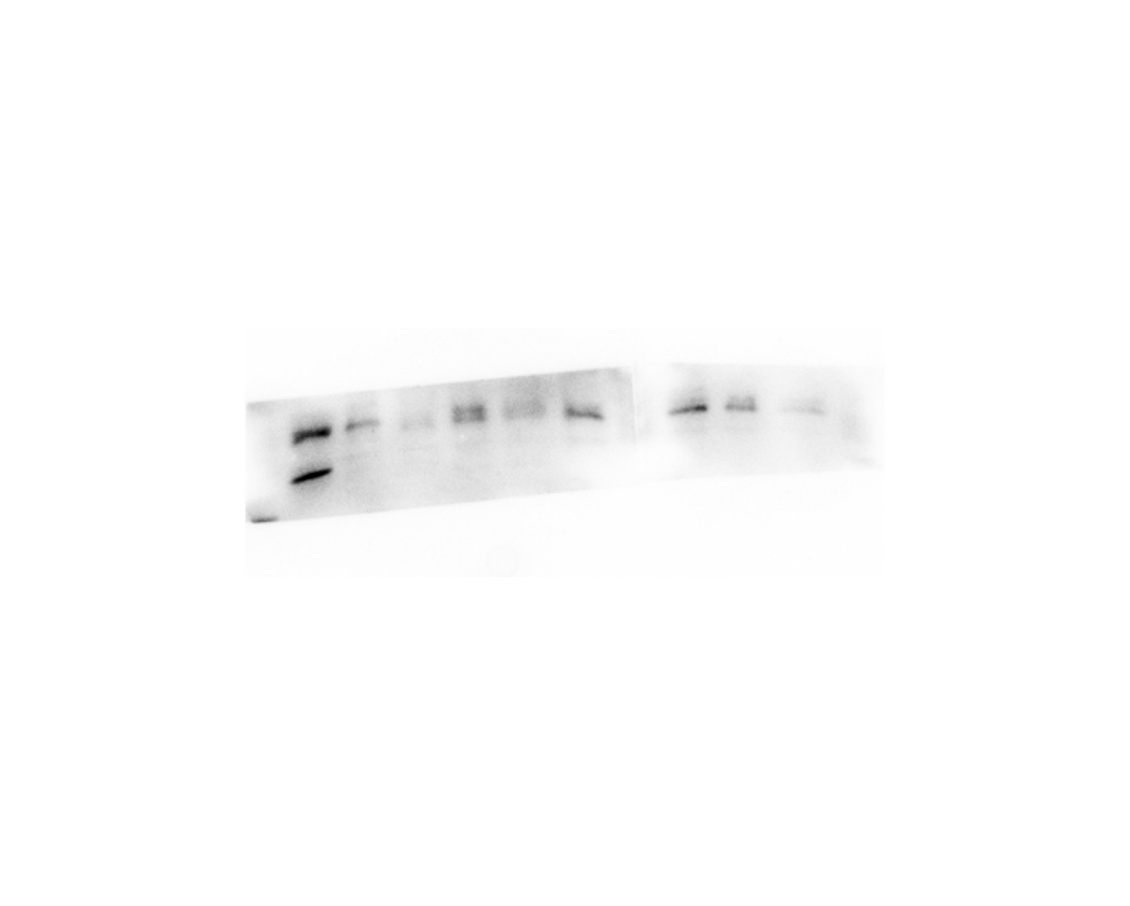

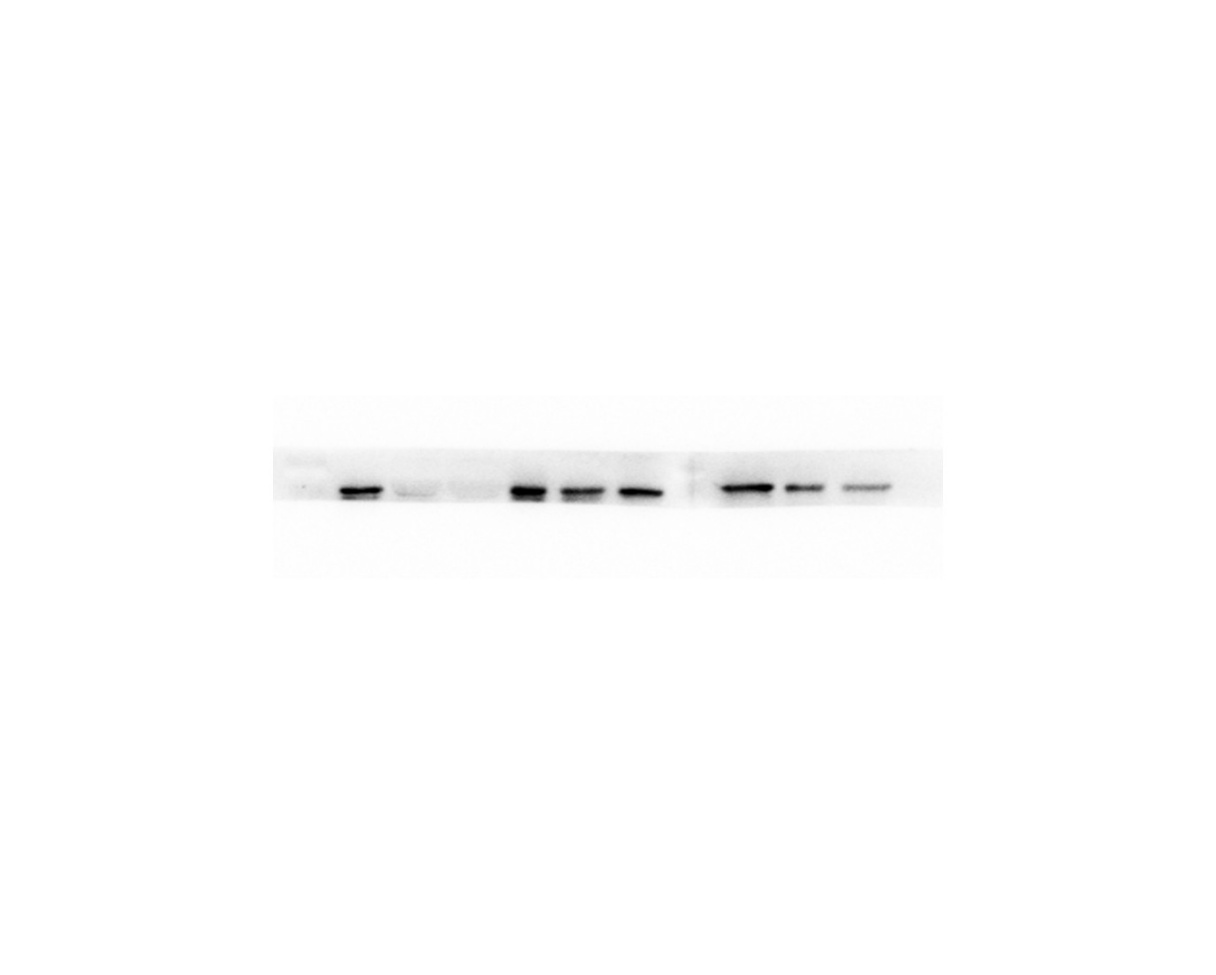


25KD

35KD

35KD

2L 05.5 95.11c

**Figure 1 panel I anti-PP2Ac**

Ladder

70KD

2L 05.5 95.11c

**Figure 1 panel I anti-PR65A**

**
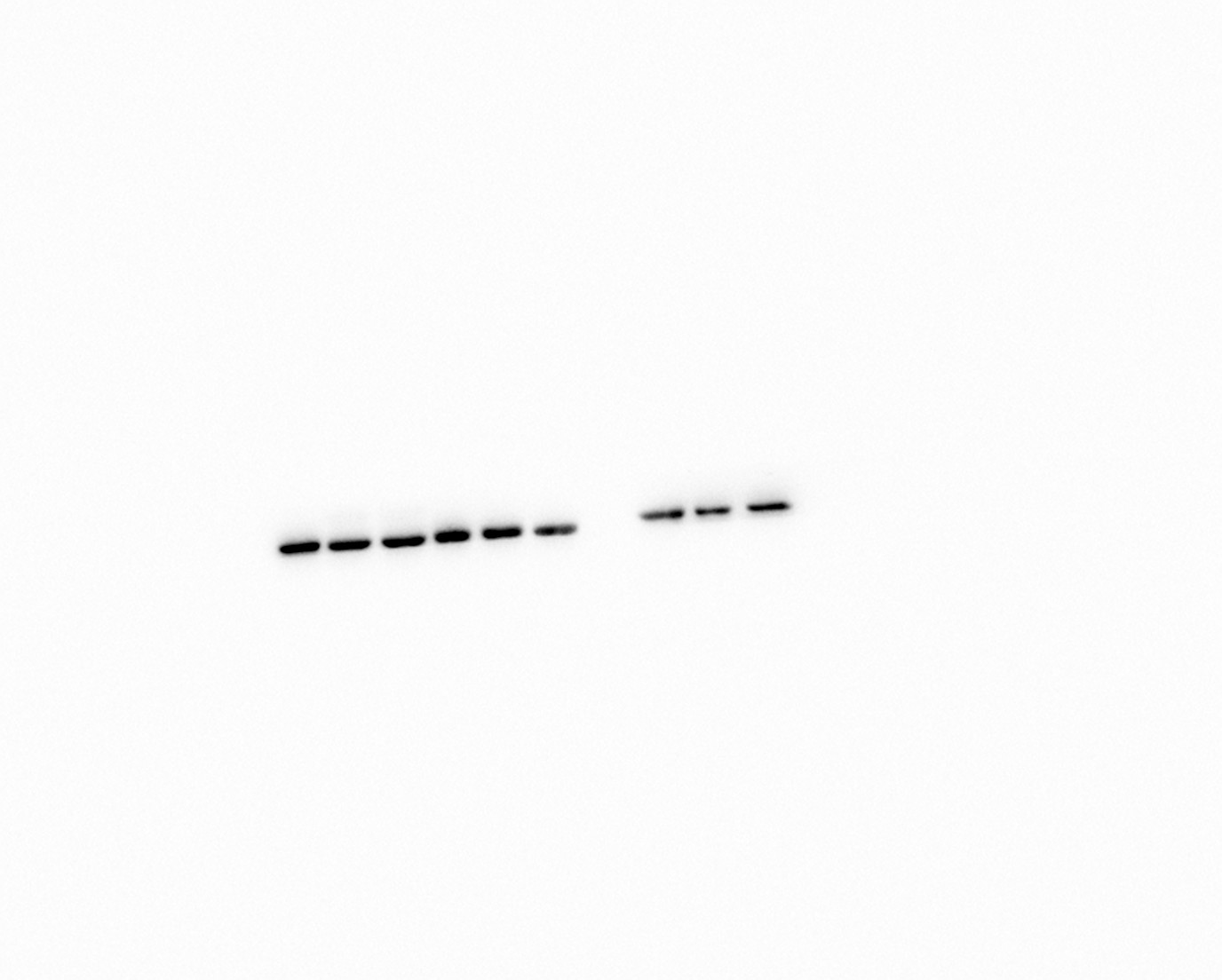
**

2L 05.5 95.11c

**Figure 1 panel I anti-Actin**
