# Supplementary material for: Decreased PP2A expression and activity represent a therapeutic target for plexiform neurofibroma
Source: Acta Neuropathol Commun. 2026 May 11;14:147. doi: 10.1186/s40478-026-02315-w (PMC13366792; doi:10.1186/s40478-026-02315-w)
Supplement: Supplementary file 2 — Supplementary Material 2. [file 40478_2026_2315_MOESM2_ESM.docx]

**Supplemental Materials**

**Decreased PP2A Expression and Activity Represent a Therapeutic Target for Plexiform Neurofibroma**

**Supplemental Table 1: Genes and Their Encoded Proteins and Subunits of PP2A**

| **Gene Name** | **Protein Name** | **Subunit** |
| --- | --- | --- |
| *PPP2R1A* | PR65-α（PR65A） | Scaffolding/Structural A subunit |
| *PPP2R1B* | PR65-β |  |
| *PPP2CA* | PP2Acα（PP2CA) | Catalytic C subunit |
| *PPP2CB* | PP2Acβ |  |
| *PPP2R2A* | B55-α | Regulatory B subunit |
| *PPP2R2B* | B55-β |  |
| *PPP2R2C* | B55-γ |  |
| *PPP2R2D* | B55-δ |  |
| *PPP2R5A* | B56-α | Regulatory B' subunit |
| *PPP2R5B* | B56-β |  |
| *PPP2R5C* | B56-γ |  |
| *PPP2R5D* | B56-δ |  |
| *PPP2R5E* | B56-ε |  |
| *PPP2R3A* | PR72/PR130 | Regulatory B'' subunit |
| *PPP2R3B* | PR70/PR48 |  |
| *PPP2R3C* | G5PR |  |
| *PPP2R6A* | STRN | Regulatory B''' subunit |
| *PPP2R6B* | STRN3 |  |
| *PPP2R6C* | STRN4 |  |

**
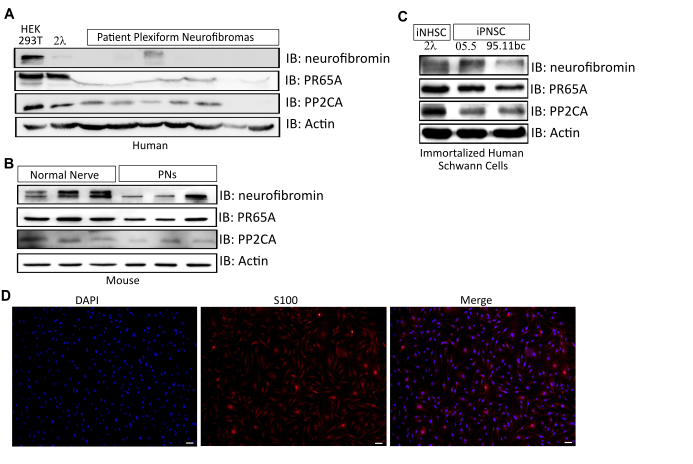
Supplemental Figure 1 *NF1*, *PPP2R1A, and PPP2CA* are downregulated in neurofibroma tumors and immortalized neurofibroma Schwann cells; S100 staining indicated the Schwann cell lineage.** Decreased expression of neurofibromin, PR65A, and PP2CA is confirmed in human neurofibroma tissues (A), mouse neurofibroma tissues (B), and immortalized neurofibroma Schwann cells (B) compared to normal nerve or normal Schwann cells by western blot. (D) Human neurofibroma-derived primary Schwann cells were stained as S100+, indicating the identity of Schwann cell lineage. Scale bar: 50μm.


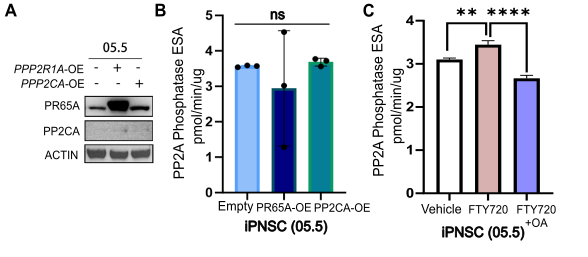


**Supplemental Figure 2 PP2A phosphatase activity regulated by PR65A/PP2CA overexpression or pharmacological inhibitor Okadaic Acid (OA). A.** Western blot of immortalized plexiform neurofibroma Schwann cells (iPNSC 05.5) with overexpression of *PPP2R1A or PPP2CA*. PR65A was successfully overexpressed, but PP2CA was not. **B.** Quantitative analysis of PP2A phosphatase activity in iPNSC (05.5) with PR65A or PP2CA overexpression. **C.** Quantitative analysis of PP2A phosphatase activity in iPNSC (05.5) with FTY720 or a combination of FTY720 and OA treatment. Data are presented as mean ± SEM; ****p < 0.0001 (by One-way ANOVA).


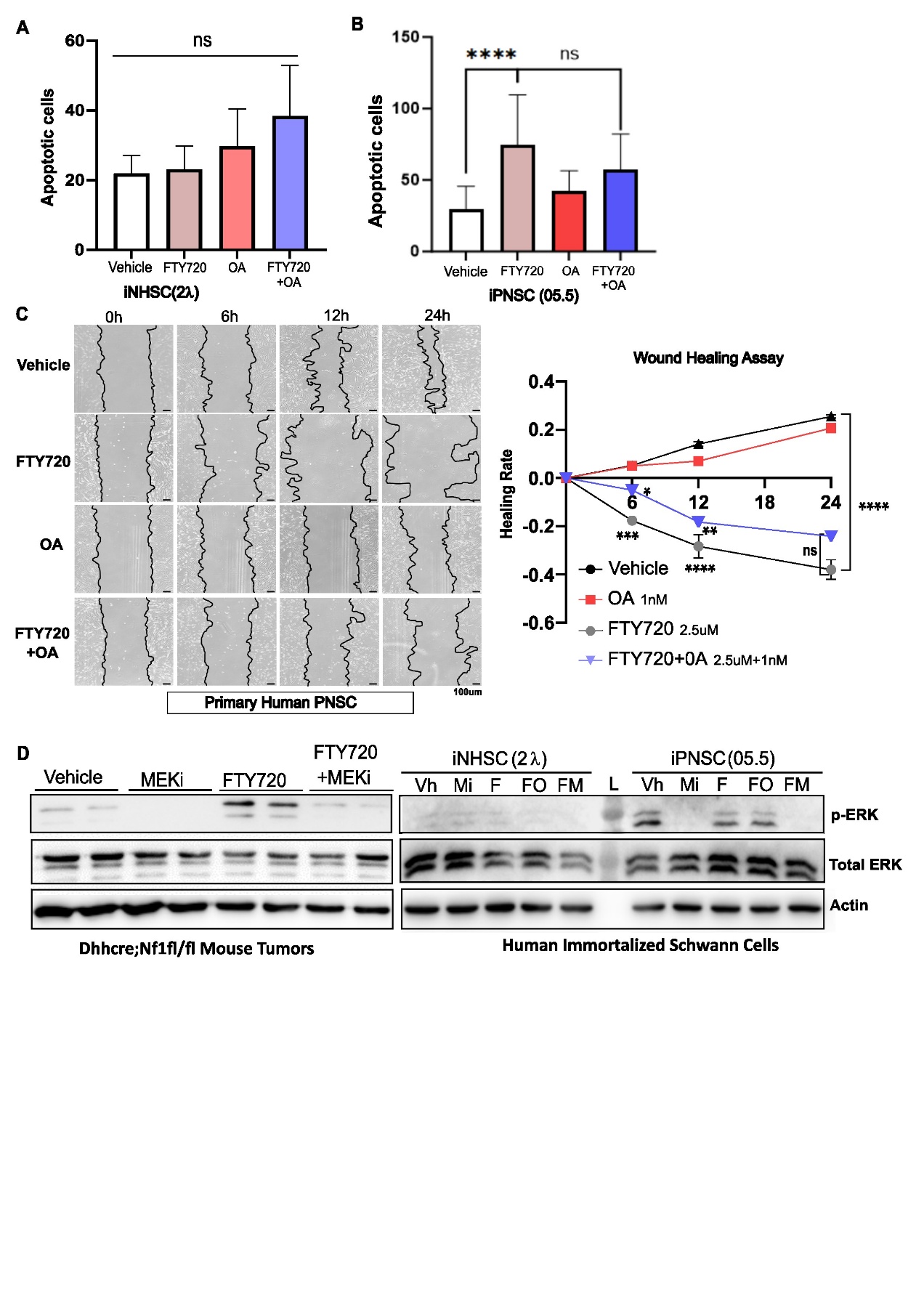


**Supplemental Figure 3 FTY720 induces iPNSC apoptosis and primary Schwann cells migration in a PP2A partially dependent manner; FTY720 induces higher p-ERK in mouse neurofibromas. A and B.** Quantitative analysis of apoptosis in iNHSC (2λ) and iPNSC (05.5) following treatment with vehicle, FTY720, OA, or FTY720 combined with OA. Apoptotic cells are AnnexinV+/PI- plus AnnexinV+/PI+ cells, and were quantified from ten randomly selected fields per treatment group following Annexin V/PI staining. Data are shown as mean ± SEM. Statistical significance was determined by one-way ANOVA. *p < 0.05, **p < 0.01, ***p < 0.05. **C.** Representative images of wound healing assay showing the migration of PNSC after treatment with vehicle, FTY720, OA, or FTY720 plus OA. Following the quantitative analysis of the wound healing assay. Three replicates were quantified for each time point. Statistical significance was determined by Two-way ANOVA. *p < 0.05, **p < 0.01, ***p < 0.001. **D.** Western blot of p-ERK in mouse neurofibroma tumors or iNHSC (2λ) and iPNSC (05.5). Mice were treated with vehicle, MEKi, FTY720, or combination of FTY720 and MEKi. Cells were treated with vehicle, MEKi (Mi), FTY720 (F), FTY720 combined with OA (FO), and FTY720 combined with MEKi (FM).


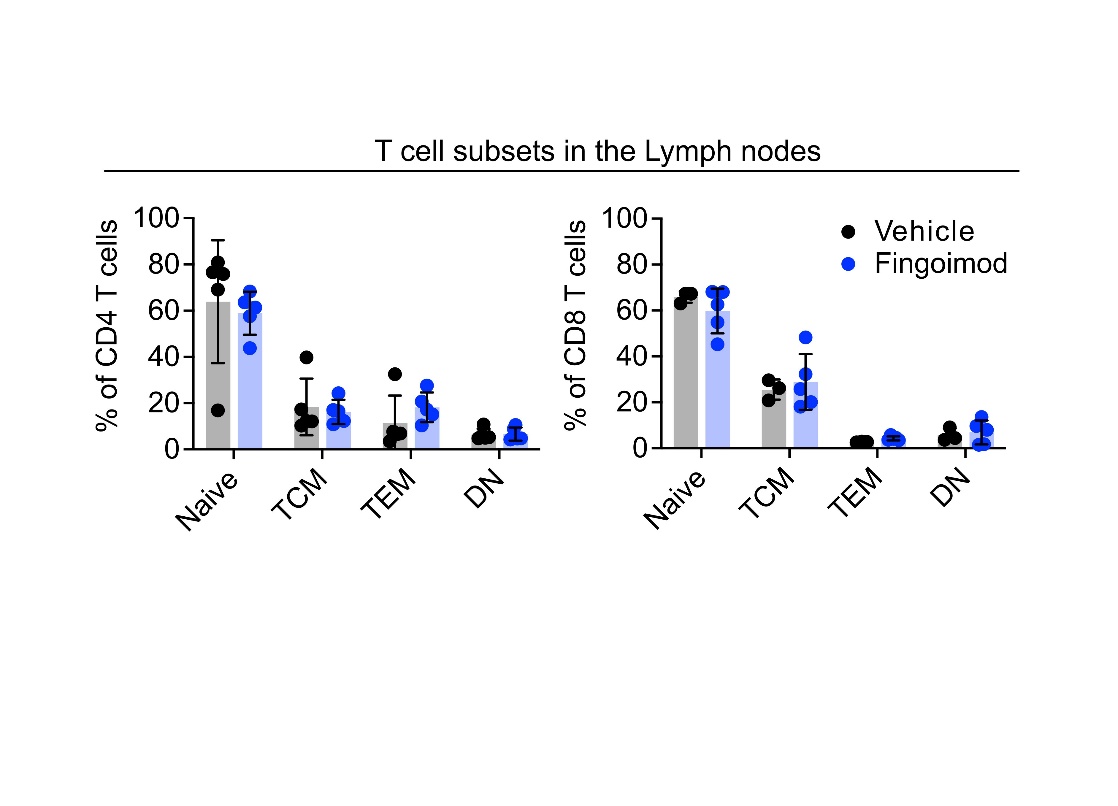


**Supplemental Figure 4. Quantification of CD4⁺ and CD8⁺ T-cell subsets in lymph nodes based on CD44 and CD62L expression.** Lymph node cells from vehicle and FTY720 treated mice were analyzed by flow cytometry. Frequency of each T-cell subset among total CD4⁺ and CD8⁺ T cells. Data are presented as mean ± SD (n = 3 Veh; n = 5 FTY720). Statistical analysis was performed using one-way ANOVA. No significant differences were observed between groups (p > 0.05).


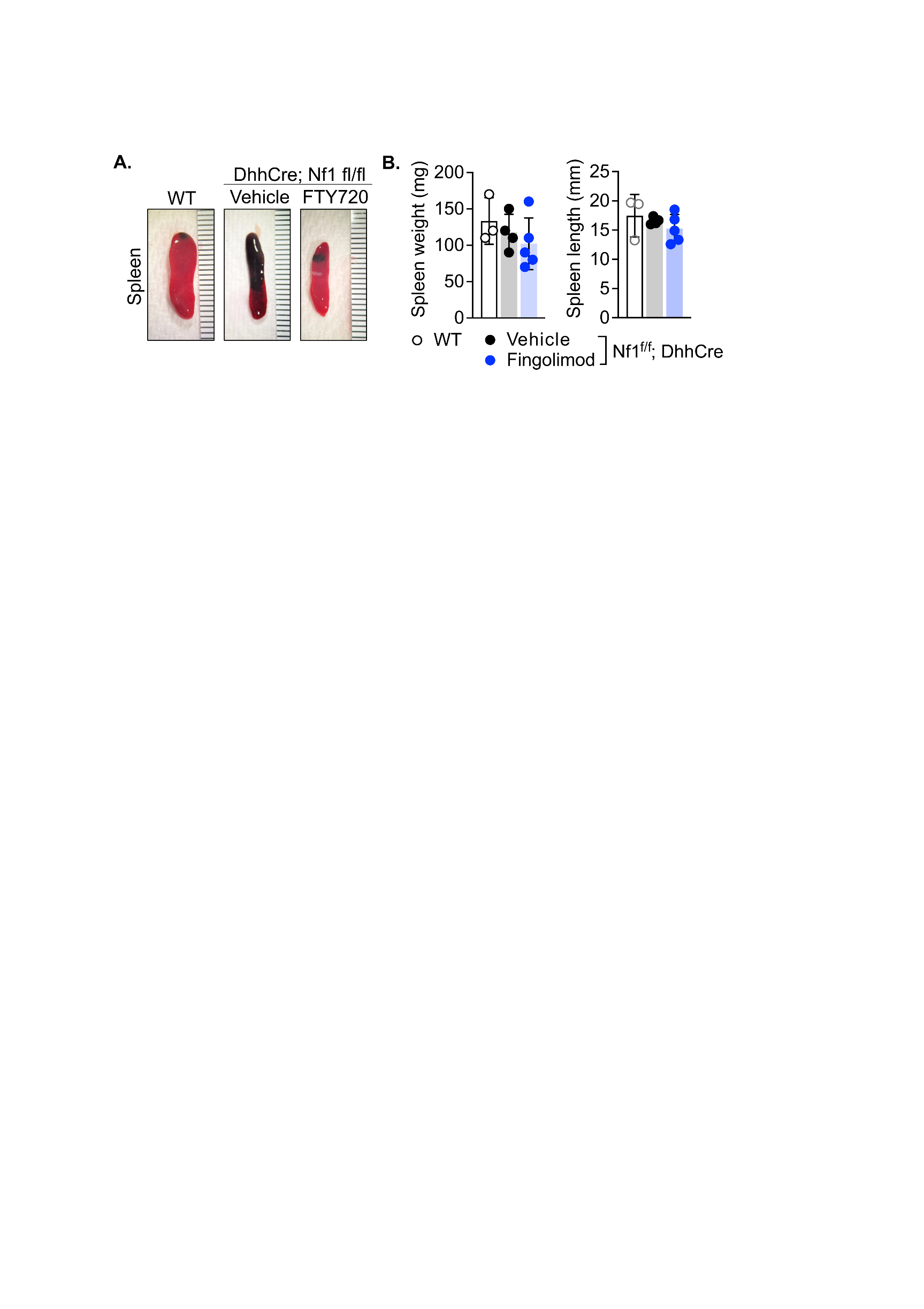


**Supplemental Figure 5. No significant difference in spleen size or weight between vehicle- and drug-treated groups.** Spleens were collected from animals treated with either vehicle or FTY720 for 30 days. **A.** Representative images of spleens from wild type or DhhCre; Nf1f/f mice treated with vehicle or FTY720. **B.** Quantification of spleen weight and length. Data are presented as mean ± SD (n = 3, WT; n= 4 Veh; n=5 FTY720). Statistical analysis was performed using an unpaired two-tailed Student’s t-test. No significant differences were observed (p > 0.05).


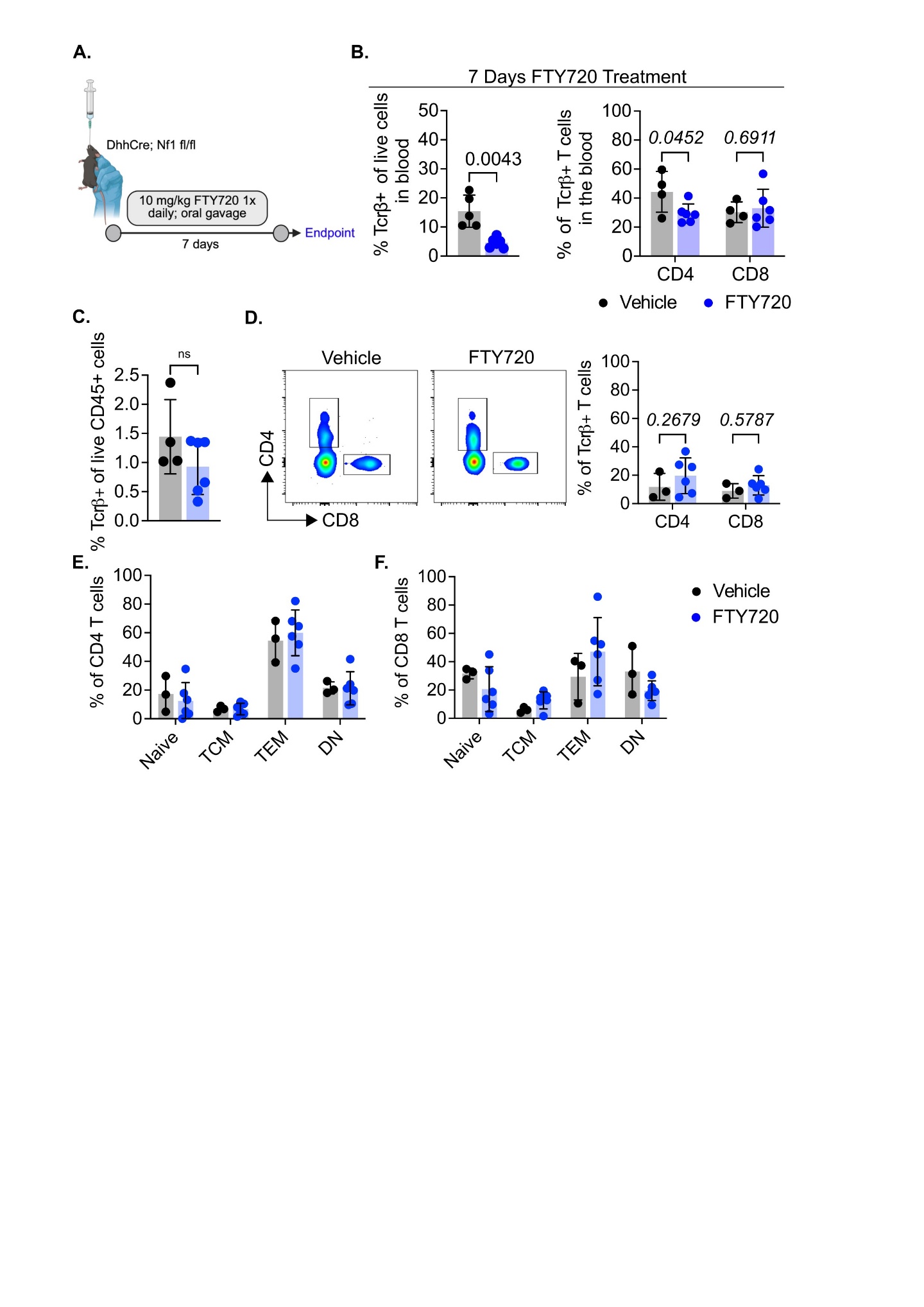


**Supplemental Figure 6. Short-term FTY720 treatment alters circulating T-cell distribution but does not impact intratumoral T-cell abundance or activation state**. **A.** Schematic representation of the experimental design. Mice were treated with FTY720 for 7 days prior to tissue collection and downstream analyses. **B.** Frequency of circulating T cells following 7 days of FTY720 treatment. Total T cells were identified as TCRβ⁺ cells, alongside CD4⁺ and CD8⁺ T-cell subsets. FTY720 treatment resulted in a reduction of circulating TCRβ⁺ T cells, with a decrease in CD4⁺ T-cell frequency and no significant change in CD8⁺ T-cell frequency. **C.** Frequency of total T cells (TCRβ⁺) within the tumor microenvironment. No significant differences were observed between control and FTY720-treated groups. **D.** Frequency of CD4⁺ and CD8⁺ T cells within tumors. FTY720 treatment did not alter the proportion of either subset in the tumor. **E.** Activation state of intratumoral CD4 and **F.** CD8 T cells, assessed by CD44 and CD62L expression. No significant differences in activation or differentiation profiles were observed between control and FTY720-treated groups. Data are presented as mean ± SD with individual data points representing biological replicates. Statistical significance was determined using ANOVA non-parametric test, with *p < 0.05 considered significant.
